# Facile Synthesis of Band Gap-Tunable Kappa-Carrageenan-Mediated C,S-Doped TiO_2_ Nanoparticles for Enhanced Dye Degradation

**DOI:** 10.1021/acsomega.4c01370

**Published:** 2024-04-29

**Authors:** Daisy Jane D. Erjeno, Dan Michael A. Asequia, Carlo Kurt F. Osorio, Christine Joy M. Omisol, Andrei E. Etom, Renzo Miguel R. Hisona, Amierson C. Tilendo, Ann Pearl G. Triana, Gerard G. Dumancas, Joshua B. Zoleta, Arnold C. Alguno, Roberto M. Malaluan, Arnold A. Lubguban

**Affiliations:** † Center for Sustainable Polymers, Mindanao State University − Iligan Institute of Technology, Iligan City 9200, Philippines; ‡ Department of Chemistry, The University of Scranton, Scranton, Pennsylvania 18510, United States; § Department of Physics, Mindanao State University − Iligan Institute of Technology, Iligan City 9200, Philippines; ∥ Department of Materials Resources Engineering and Technology, Mindanao State University − Iligan Institute of Technology, Iligan City, 9200 Philippines; ⊥ Chemical Engineering Department, Mindanao State University − Marawi, Marawi City 9700, Philippines

## Abstract

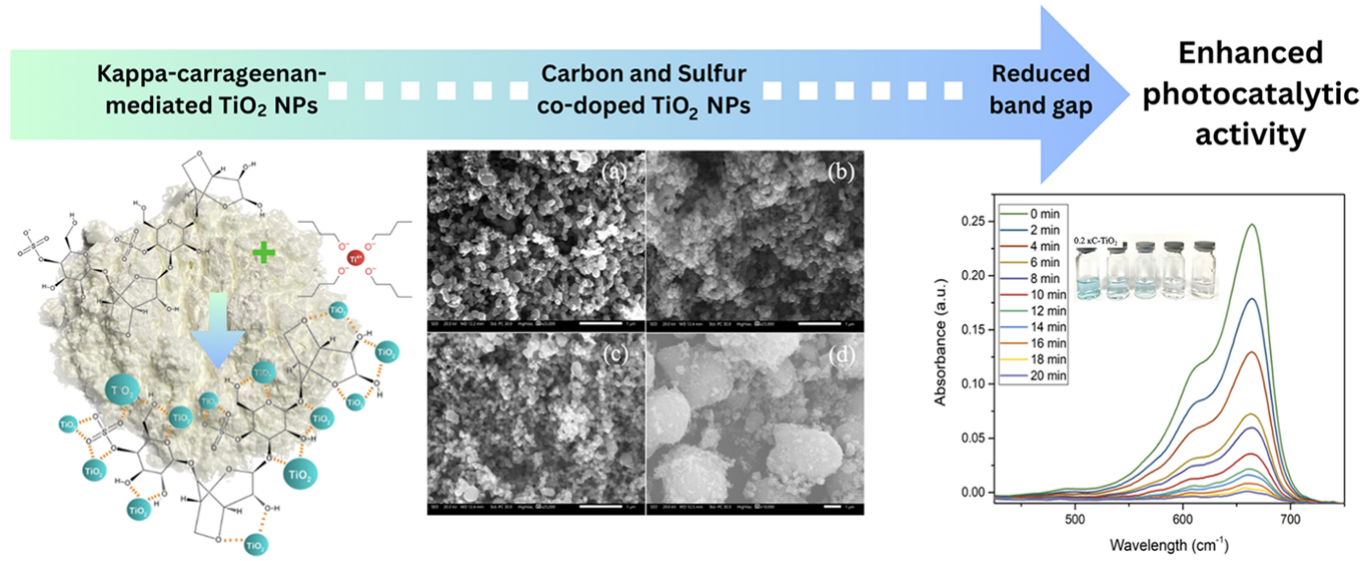

Semiconducting nanoparticles (SNPs) have garnered significant attention for their role in photocatalysis technology, offering a cost-effective and highly efficient method for breaking down organic dyes. Of particular significance within SNP-based photocatalysis are tunable band gap TiO_2_ nanoparticles (NPs), which demonstrate remarkable enhancement in photocatalytic efficiency. In the present work, we introduce an approach for the synthesis of TiO_2_ NPs using kappa-carrageenan (κ-carrageenan), not just as a reducing and stabilizing agent but as a dopant for the resulting TiO_2_ NPs. During the synthesis of TiO_2_ NPs in the presence of sulfate-rich carrageenan, the process predominantly leaves residual sulfur and carbon. The presence of residual carbon, in conjunction with sulfur doping, as indicated by fast FTIR spectra, XPS, and EDX, leads to a significant reduction in the band gap of the resulting composite to 2.71 eV. The reduction of composite band gap yields remarkable degradation of methylene blue (99.97%) and methyl orange (97.84%). This work presents an eco-friendly and highly effective solution for the swift removal of environmentally harmful organic dyes.

## Introduction

An alarming 80% of the global population faces exposure to severe water pollution, marking a critical environmental challenge in the 21st century.^1^ The textile and pharmaceutical sectors, utilizing metals and synthetic organic/inorganic compounds, pose significant risks to life. The wastewater discharged by these textile industries is frequently heavily colored with synthetic dyes and chemicals, posing severe health risks to humans and ecological harm to plants, animals, and microorganisms.^2,3^ This highly colored textile wastewater, primarily containing heavy metals, azo dyes, and other toxic substances, can disrupt essential biochemical processes like photosynthesis in plants and even lead to problems like eutrophication in aquatic environments.^4^ For instance, azo dyes, such as methylene blue (MB) and methyl orange (MO), make up around 70% of the commercial dyes utilized in the global textile industry.^5^ Approximately 50% of the synthetic dyes utilized in textile sectors are purportedly nonadherent to fabric substrates, thereby facilitating their leaching into the surrounding environment. This exposure introduces a broad spectrum of chemical and microbiological pollutants into aquatic ecosystems.^6,7^ These azo dyes are also known for their high toxicity and carcinogenic properties.^5^ Therefore, addressing the environmental impact of these pollutants is crucial for sustainable industrial practices and water quality preservation.^8^

Several methods are employed to remove harmful dyes from industrial wastewater, including coagulation, adsorption, biodegradation, membrane processes, activated sludge treatment processes (ASTP), and advanced oxidation processes (AOP).^9−11^ For instance, ASTPs are commonly used in wastewater treatment plants due to their cost-effectiveness but are ineffective in removing hazardous organic dyes.^12^ Conversely, physicochemical treatments like coagulation and adsorption might raise environmental concerns due to their use of chemicals, high energy intensity, generation of sludge, and special resource requirements, which can have adverse ecological effects.^13^ On the other hand, membrane processes, such as membrane bioreactors, achieve high-quality water treatment through effective physical separation of solids and microorganisms but are energy-inefficient and expensive to operate.^13,14^ AOPs, however, hold great promise for treating textile wastewater containing dyes due to their capability to effectively break down soluble organic pollutants.^15,16^ Furthermore, heterogeneous photocatalytic degradation-based AOPS utilizes low-cost semiconductor-based photocatalysts, thus offering a more cost-effective and highly efficient method for breaking down organic dyes compared with other AOPS.^17^

Semiconducting nanoparticle (SNP)-based photocatalysis exhibits substantial potential for heterogeneous photocatalytic degradation-based AOPs owing to its simplicity, cost-effectiveness, nontoxic nature, impressive degradation efficiency, and outstanding stability.^18^ Numerous SNPs, including ZnO, CuO, TiO_2_, NiO, and SnO_2,_ have been harnessed as photocatalysts to eliminate organic dyes.^19−23^ Among these, titanium dioxide (TiO_2_) has garnered significant attention in photocatalysis.^22^ In 1972, Fujishima and Honda first suggested employing TiO_2_ in photocatalysis, illustrating its capability to split water and generate hydrogen under light exposure.^24^ Subsequently, extensive research has been conducted on TiO_2_ photocatalysis, catalyzing advancements in water^25^ and air purification^26^ technologies. Utilizing photocatalytic oxidation, this approach has demonstrated practicality in sewage treatment and air purification by decomposing pollutants and transforming organic substances into minerals, thus preventing further pollution.^27^ TiO_2_ photocatalysts, among numerous photocatalytic semiconductor options, have garnered extensive research and application for producing hydrogen via photocatalysis and photodegradation applications. This is due to their exceptional attributes such as abundant availability, chemical durability, strong catalytic capability, resistance to photocorrosion, and nontoxic nature.^28^

Despite the remarkable catalytic efficacy of TiO_2_, indicated by a band gap energy ranging from 3.0 to 3.2 eV, its limited solar energy absorption poses a challenge for efficient photocatalysis.^29^ This limitation arises due to the critical role of the electronic band structure and the band gap energy in determining the effectiveness of a photocatalyst. For optimal performance, the band gap energy should ideally be below 3 eV to facilitate broadened light absorption into the visible range and maximize solar energy utilization.^30^ Consequently, the implementation of strategies becomes imperative to enhance TiO_2_’s photocatalytic activity. Researchers commonly use surface modification methods to bolster TiO_2_’s adsorption capacity and reduce its band gap. These methods typically involve doping specific species or incorporating metal/nonmetal dopants into the TiO_2_ structure.

Specifically, nonmetal doping with elements like nitrogen, carbon, sulfur, fluorine, or iodine has proven effective in reducing the band gap and improving photocatalytic activity.^31^ Doping TiO_2_ with nonmetal elements modifies its electronic band structure, reducing the band gap energy and enhancing responsiveness to solar energy.^32^ Sulfur and carbon-doped TiO_2_, mainly, have garnered attention for their ability to decrease the band gap, highlighting significant potential for photocatalytic applications.^33,34^

Carrageenan, derived from red seaweeds (*Rhodophyta*), consists of water-soluble polysaccharides and is a thickening ingredient in the food industry.^35^ Its notable gel-forming properties, from a high water-absorption capacity, make it conducive to constructing biohydrogels.^36^ Additionally, carrageenan hydrogels have served as an eco-friendly agent for reducing and stabilizing NPs under ultrasonic irradiation, with studies highlighting their effectiveness in controlling the size and shape of NPs.^37^ Comprising galactose and anhydrogalactose units connected by glycosidic bonds, carrageenans also feature a matrix rich in carbon and sulfur.^38^ Hence, carrageenan has emerged as a promising material, serving as a reducing and stabilizing agent for NP synthesis and as a source for carbon and sulfur as nonmetal dopants in TiO_2_. This leads to a reduced band gap and the production of highly active photocatalysts.

Chaudhary et al. initially demonstrated the application of various sulfate-rich carrageenans as direct sources for sulfur and carbon doping in TiO_2_, resulting in a highly active photocatalyst. The study further elucidated the impact of these seaweed carrageenans, specifically kappa, iota, and lambda, on the photodegradation of industrially essential dyes.^39^ While carrageenan has previously been utilized for synthesizing TiO_2_ NPs for dye photocatalytic degradation, its potential for tuning the band gap energy of TiO_2_ remains unreported. In this investigation, κ-carrageenan exhibited multifunctional properties by acting as a reducing and stabilizing agent and serving as a carbon and sulfur source for the doping process of TiO_2_ nanoparticles. This diverse role reduced the band gap of the resulting nanoparticles, thereby enhancing its photocatalytic activity. The synthesized C, S doped TiO_2_ nanoparticles (κC-TiO_2_ NPs) underwent evaluation for their efficacy in degrading methylene blue (MB) and methyl orange (MO) in an aqueous medium, indicating a promising avenue for mitigating environmental contaminants present in wastewater.

## Results and Discussion

This research aimed to employ κ-carrageenan not only as a reducing and stabilizing agent but also as a source of carbon and sulfur for doping TiO_2_ NPs, resulting in a reduced band gap and enhanced photocatalytic activity. To explore the impact of varying κ-carrageenan concentrations on the band gap energy of κC-TiO_2_ NPs, three samples were produced at 0.1%, 0.2%, and 0.3% (w/v), designated as 0.1 κC-TiO_2_ NPs, 0.2 κC-TiO_2_ NPs, and 0.3 κC-TiO_2_ NPs, respectively. Subsequently, the κC-TiO_2_ NPs were assessed for their photocatalytic activity against MB and MO dyes and compared to commercially available pure TiO_2_ NPs.

### Powder XRD Analysis

**Figure 1:**
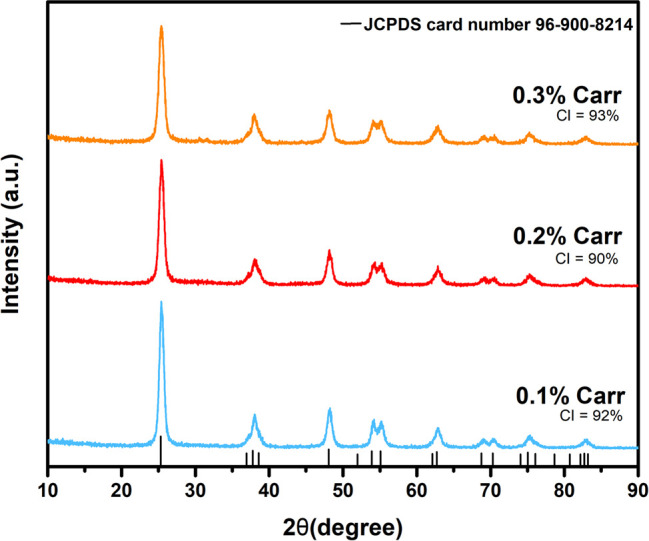
XRD patterns of TiO_2_ nanoparticles synthesized using various concentrations of κ-carrageenan (κC-TiO_2_ NPs).

XRD analysis was conducted to investigate the crystalline characteristics of produced κC-TiO_2_ NPs, as shown in Figure 1. The established XRD peaks in all three samples observed at 2θ values of 25.3°, 38.6°, 48.1°, 53.9°, 55.1°, 62.7°, 68.8°, 70.4°, 75.1°, and 82.2°, align with the crystallographic planes (101), (112), (200), (105), (211), (204), (116), (220), (215), and (303), respectively. The identified peaks closely mirror the characteristic tetragonal configuration associated with anatase TiO_2_, as delineated in the JCPDS Card No. 96–900–8214. Figure 1 demonstrates that all peaks align with JCPDS Card No. 96–900–8214, confirming the absence of contaminants or mismatched peaks.^40^

The NPs’ average crystallite sizes were determined using Debye–Scherrer’s equation:
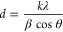
1where *d* represents the mean diameter of the crystalline domains, λ denotes the wavelength, and β indicates the angular peak at the diffraction angle θ.

No observable diffraction patterns indicate the presence of carbon, sulfur, or other phases, suggesting that carbon and sulfur have been fully incorporated into the TiO_2_ structure.^41,42^ Results demonstrate a decrease in crystallite size with increasing κ-carrageenan concentrations. The mean crystallite sizes were calculated to be 9.2, 8.7, and 8.0 nm for TiO_2_ NPs synthesized with 0.1%, 0.2%, and 0.3% κ-carrageenan concentrations, respectively. These findings affirm that κ-carrageenan facilitates the creation of a highly crystalline anatase phase of TiO_2_ NPs. High crystalline indices (CI) of 92%, 90%, and 93% were calculated for 0.1%, 0.2%, and 0.3% concentrations, respectively, underscoring the pronounced crystallinity of the synthesized κC-TiO_2_ NPs. The distinct XRD peaks observed in the case of κC-TiO_2_ NPs indicate their distinctly high crystalline nature, which contributes to the improvement in the photocatalytic performance of the material. This can be explained by the improved movement of electrons from the conduction band of TiO_2_ to the molecules adsorbed on the surface.^43^

### UV–Visible Spectroscopic Analysis

**Figure 2:**
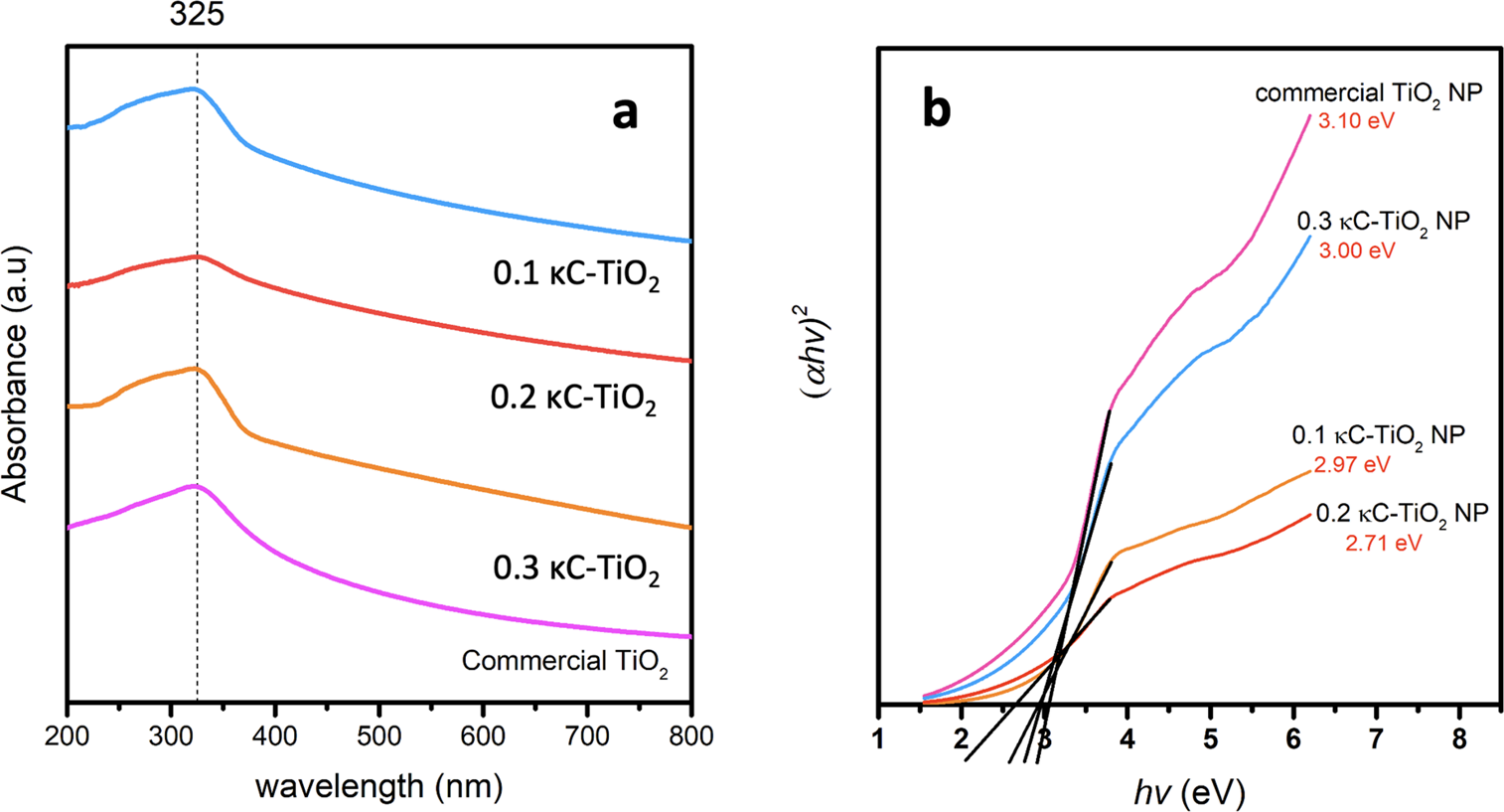
(a) UV–vis spectroscopy absorption spectra and (b) band gap energy of TiO_2_ nanoparticles synthesized with varying concentrations of κ-carrageenan (κC-TiO_2_ NPs) compared with pure commercial TiO_2_ NPs.

The light absorption characteristics of the *κC-*TiO_2_ NPs synthesized at varying concentrations of κ-carrageenan were assessed by UV–vis spectra analysis across a wavelength range from 200 to 800 nm. This assessment was conducted using a suspension of *κC-*TiO_2_ NPs in distilled water. The UV–vis analysis indicated a peak in absorption at 325 nm, as illustrated in Figure 2a. This absorption peak closely corresponds to the UV–vis band observed at 320 nm in TiO_2_ NPs, as reported by Devikala and Abisharani.^44^

The absorption coefficient of TiO_2_ NPs was calculated using eq 2:
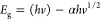
2where α is the absorption coefficient, *hv* is the photon energy, and E_g_ is the optical band gap energy. The E_g_ for both commercial TiO_2_ NPs and the synthesized κC-TiO_2_ NPs at different κ-carrageenan concentrations is calculated using the Tauc plot shown in Figure 2b. This involves plotting *αhv*^1/2^ against *hv*. The E_g_ values for commercial TiO_2_ NPs and κC-TiO_2_ NPs synthesized with carrageenan at varying concentrations (0.1%, 0.2%, and 0.3%) are determined as 3.10, 2.97, 2.71, and 3.00 eV, respectively. These findings closely align with previously reported results, particularly in studies involving Kondagogu gum-mediated TiO_2_ NPs, where the E_g_ ranged from 2.99 to 3.18 eV.^45^

Figure 2b also illustrates that κC-TiO_2_ NPs synthesized with κ-carrageenan exhibit comparatively lower E_g_ values than pure commercial TiO_2_ NPs. This observed change is attributed to residual carbon and sulfur from κ-carrageenan, which are incorporated onto the surface of the κC-TiO_2_ NPs. Previous studies have demonstrated that the introduction of carbon and sulfur to TiO_2_ NPs can result in a decrease in its E_g_.^33,34^ The decrease in E_g_ is acknowledged for its significant benefits in boosting the photocatalytic performance of TiO_2_ nanoparticles.^46^ Furthermore, a decrease in E_g_ from 3.00 to 2.97 eV is evident as the κ-carrageenan concentration rises from 0.1% to 0.2%. However, as the κ-carrageenan concentration is further increased to 0.3%, there is a subsequent increase in E_g_. This observation is attributed to the likely saturation point reached at 0.2% κ-carrageenan concentration.

### FT-IR Analysis

**Figure 3:**
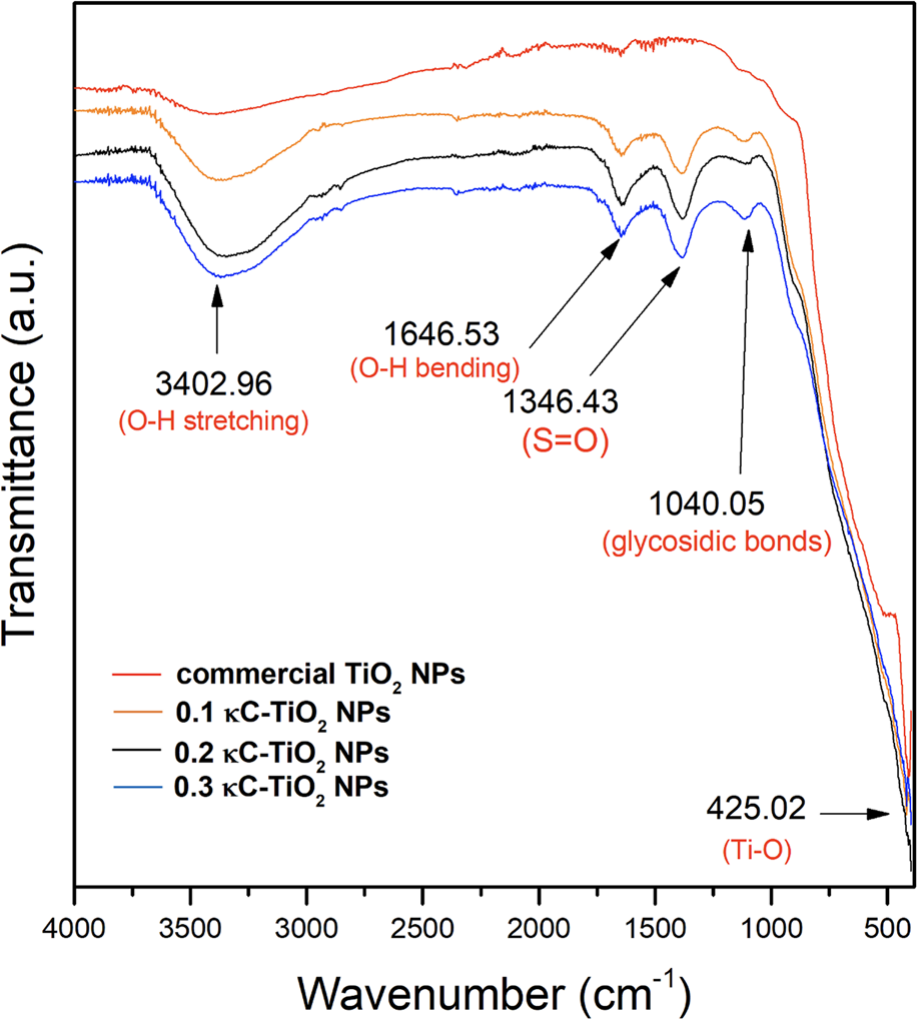
FT-IR spectra of TiO_2_ nanoparticles synthesized using different concentrations of κ-carrageenan (κC-TiO_2_ NPs) compared with pure commercial TiO_2_ NPs.

The FT-IR spectra of κC-TiO_2_ NPs and pure commercial TiO_2_ NPs in the 400–4000 cm^–1^ region are displayed in Figure 3. The peaks at approximately 3402.96 and 1646.53 cm^–1^ are ascribed to surface-absorbed water on the samples. These prominent bands emanate from the O–H stretching and bending vibrations of the chemisorbed/physisorbed H_2_O molecules on the photocatalyst surface across all samples. The intensity of these peaks is relatively greater in *κC-*TiO_2_ NP samples than in pure commercial TiO_2_ NPs. This variation in peak intensity can be explained by the charge imbalance that occurs due to the surplus of charge upon substituting Ti ions with carbon and sulfur ions in the TiO2 lattice..^47,48^ This imbalance, in turn, draws additional hydroxide ions on the surface of the photocatalyst, leading to the formation of highly reactive surface-adsorbed OH. The reduction and stabilization of *κC-*TiO_2_ NPs were evidenced by prominent absorption bands observed at 1040.05 and 1346.53 cm^–1^. Specifically, the existence of vibrations attributed to the Ti–O–S bond is indicated by a peak detected at 1040.05 cm^–1^, confirming the integration of sulfur into the lattice structure of TiO_2_.^49^ Additionally, the absorption at 1040.05 cm^–1^ may also indicate the presence of glycosidic linkage, providing further confirmation of the integration of carbon on the surface of κC-TiO_2_ NPs.^50^ The appearance of a peak around 1346.53 cm^–1^ signifies the asymmetric stretching frequency of the S=O bond, indicative of the highest oxidation state of sulfur.^51^ This finding aligns with existing literature suggesting that the catalyst with sulfate exhibits both Bronsted and Lewis acidic sites, which are crucial for its photocatalytic activity.^52^ Hence, the two distinctive bands at 1346.53 and 1040.05 cm^–1^ collectively characterize the features of sulfur and carbon doping on the surface of κC-TiO_2_ NPs.^53^ Additionally, the strong band observed in the 400–800 cm^–1^ range is attributed to the stretching vibrations of Ti–O and Ti–O–Ti bonds.

### XPS Analysis

**Figure 4:**
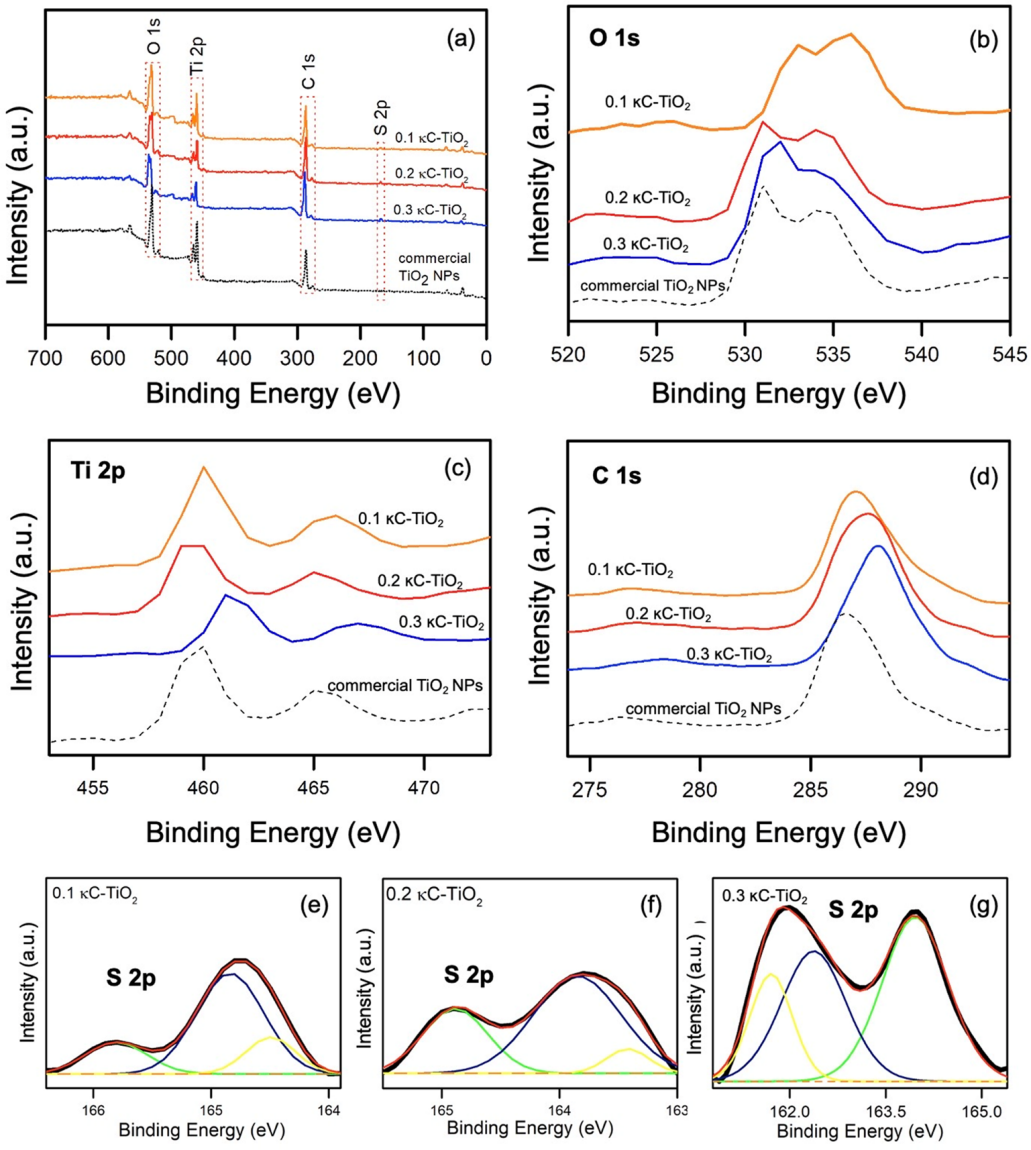
X-ray photoelectron spectroscopy (XPS) analysis illustrating (a) survey scan, (b) O 1s, (c) Ti 2p, and (d) C 1s spectra of varying concentrations of kappa-carrageenan-mediated TiO_2_ nanoparticles (0.1%, 0.2%, and 0.3%) in comparison with commercial TiO_2_ NPs. Additionally, (e–g) display S 2p region spectra of κC-TiO_2_ NPs with kappa-carrageenan concentrations of 0.1%, 0.2%, and 0.3%, respectively.

Figures 4 present the X-ray photospectrometric analysis profile of κ-carrageenan-mediated TiO_2_ nanoparticles (a) XPS wide scan, (b) O 1s narrow scan, (c) Ti 2p narrow scan, (d) C 1s narrow scan, (e) S 2p region spectra of 0.1 κC-TiO_2_, (f) S 2p 0.2 κC-TiO_2_ narrow scan, and (g) S 2p 0.3 κC-TiO_2_ narrow scan. The results revealed that XPS wide spectra of all synthesized κC-TiO_2_ could detect the presence of oxygen, carbon, titanium, and sulfur peaks at energy levels: O 1s, C 1s, Ti 2p, and S 2p, respectively.

O 1s energy level narrow scan detected two major peaks at binding energy centered at 531.4 eV and 529.1, ascribed to surface OH and Ti–O species, respectively, as shown in Figure 4b.^54^ Also, the Ti 2p narrow scan detected two major peaks centered at 458.1 and 464.5 eV and assigned to be the binding energy of Ti^3+^ 2p_3/2_ and Ti^4+^ 2p_1/2_, respectively, as shown in Figure 4c.^54^ Significant carbon C 1s peaks were detected compared to commercial TiO_2_ NPs, as shown in Figure 4d. It can be observed that the carbon peaks of κC-TiO_2_ NPs slightly shifted to higher binding energies detected at ∼287 eV.^39^ Furthermore, Figure 4(e-g) shows the narrow deconvoluted sulfur peaks at S 2p energy levels. It can be observed that all κC-TiO_2_ obtained three (3) major peaks centered at binding energy range 164.4, 165.1, and 165.8 eV assigned to the formation of S^0^, S–C, and C–SO_*x*_, respectively.^39,55,56^ This research further suggests that EDX and XPS spectroscopic analysis can confirm sulfur and carbon doping using κ-carrageenan.

### BET-BJH Analysis

**Figure 5:**
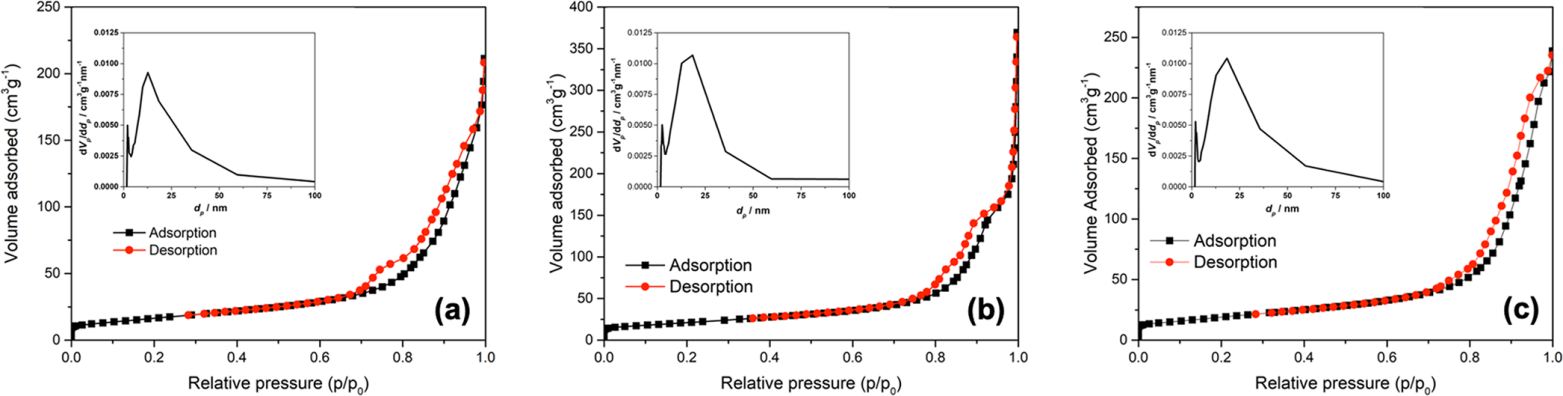
Nitrogen adsorption–desorption isotherms, and insets are the corresponding pore size distribution of (a) 0.1 κC-TiO_2_, (b) 0.2 κC-TiO_2_, and (c) 0.3 κC-TiO_2_ NPs.

The specific surface area and pore structure of commercial TiO_2_ NPs and the synthesized κC-TiO_2_ NPs were analyzed using N_2_ adsorption–desorption techniques. Previous studies have highlighted that higher specific surface areas enhance photocatalytic activity.^57^ In Figures 5a, b, and c, the N_2_ adsorption–desorption isotherms and corresponding pore size distribution of the synthesized curves of the synthesized κC-TiO_2_ NPs are depicted. All samples exhibit similar sorption isotherms and pore size distributions. The specific surface area, pore volume, and pore size of the various samples are summarized in Table 1. The 0.2 κC-TiO_2_ NPs demonstrate the highest BET surface area and BJH pore volume, suggesting an abundance of active sites conducive to efficient photocatalysis.

**Table 1:** Surface Area, Pore Size, and Pore Volume of 0.1 κC-TiO_2_, 0.2 κC-TiO_2_, 0.3 κC-TiO_2_, and Commercial TiO_2_ NPs

	BET–Surface Area	Pore Size	Pore Volume
Sample	m^2^/g	nm	cm^3^/g
0.1 κC-TiO_2_	70.38	18.23	0.54
0.2 κC-TiO_2_	75.71	18.39	0.64
0.3 κC-TiO_2_	65.20	12.70	0.34
Commercial TiO_2_ NPs	49.48	9.97	0.32

### Morphological Analysis

**Figure 6:**
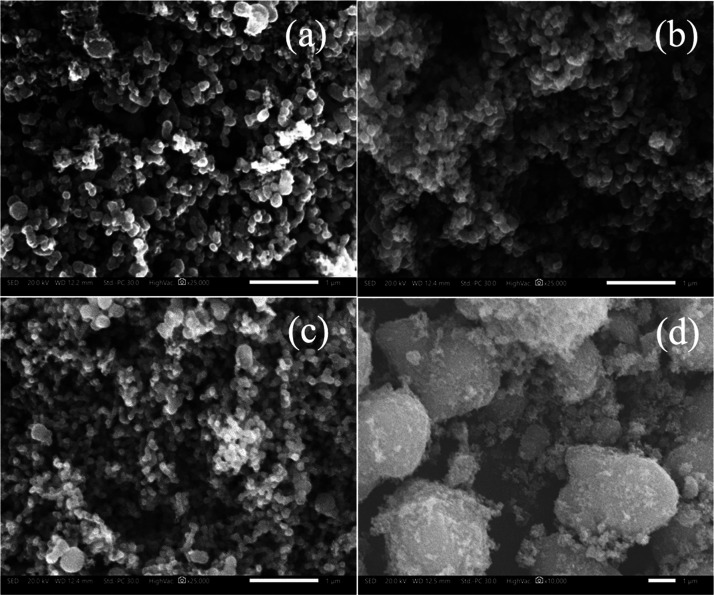
SEM images of TiO_2_ nanoparticles synthesized using different concentrations of κ-carrageenan (κC-TiO_2_ NPs) at (a) 0.1%, (b) 0.2%, and (c) 0.3% compared with (d) pure commercial TiO_2_ NPs.

After confirming the reduction, stabilization, and doping facilitated by κ-carrageenan, κC-TiO_2_ NPs underwent microscopic characterization employing SEM and TEM techniques. SEM images for κC-TiO_2_ NPs at varying κ-carrageenan concentrations are presented in Figures 6a, b, and c. These images depict the spherical structure and the spherical morphology of the synthesized κC-TiO_2_ NPs. Specifically, Figure 6a displays the formation of 0.1 κC-TiO_2_ NPs with a larger particle size, whereas Figure 6b depicts 0.2 κC-TiO_2_ NPs with decreased particle size compared with 0.1 κC-TiO_2_ NPs. These observations align with the XRD data in Figure 1. The smaller particles are also prevalent in 0.3 κC-TiO_2_ NPs (Figure 4c) but with the distinct presence of larger particles, thus broadening the particle size distribution. This phenomenon can be attributed to the excess κ-carrageenan functional groups interacting with one another rather than with the Ti ions, thus causing particle aggregation and increasing particle sizes.^37^

**Figure 7:**
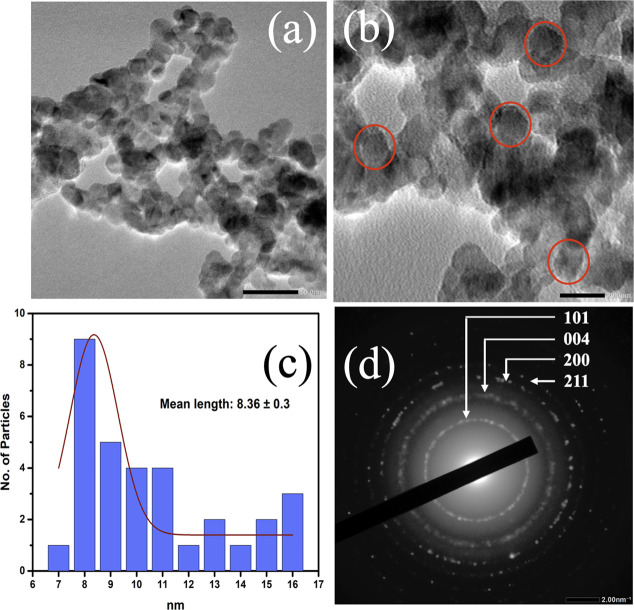
TEM images of spherical-shaped nanocrystalline titanium dioxide nanoparticles (κC-TiO_2_ NPs) at 0.2% concentrations of kappa-carrageenan under (a) 50 nm magnification, (b) 20 nm magnification, (c) size distribution, and (d) SAED.

Notably, 0.2% κC-TiO_2_ NPs exhibit the most uniformly distributed particle size among the three synthesized samples. This property can be attributed to the sufficiently homogeneous distribution of OH, sulfate, and glycosidic groups along the κ-carrageenan chain.^37^ The mentioned structural feature of κ-carrageenan is conducive to the efficient donation of electrons required to reduce, stabilize, and dope TiO_2_ during NP synthesis. This observation corresponds with the results reported in the investigation conducted by Wan et al. in 2021, highlighting the significance of κ-carrageenan concentration in producing silver nanoparticles that are both small and uniform.^37^ Furthermore, the uniformity in the size and morphology of κC-TiO_2_ NPs plays a crucial role in photodegradation processes.^58^ Smaller and uniformly sized TiO_2_ NP photocatalysts have advantages in various aspects of photocatalysis.^59,60^ They possess higher light absorption efficiency due to a larger surface area that enhances the initiation of photodegradation reactions,^61^ and exhibit better stability in photocatalysis, showing reduced tendencies for aggregation and sedimentation, thereby prolonging the catalyst’s performance.^62^ Hence, the selection of 0.2% κC-TiO_2_ NPs for the ensuing photocatalytic experiments was based on its advantageous attributes of small particle size and uniformity. Moreover, Figure 7 provides TEM images and particle size distribution of the κ-carrageenan-derived TiO_2_ (0.2% κC-TiO_2_) NPs. The TEM analysis unveils nanocrystalline TiO_2_ NPs with spherical shapes and grain sizes ranging from 7 to 16 nm. These grain sizes agree with the conclusions drawn from the XRD data.

**Figure 8:**
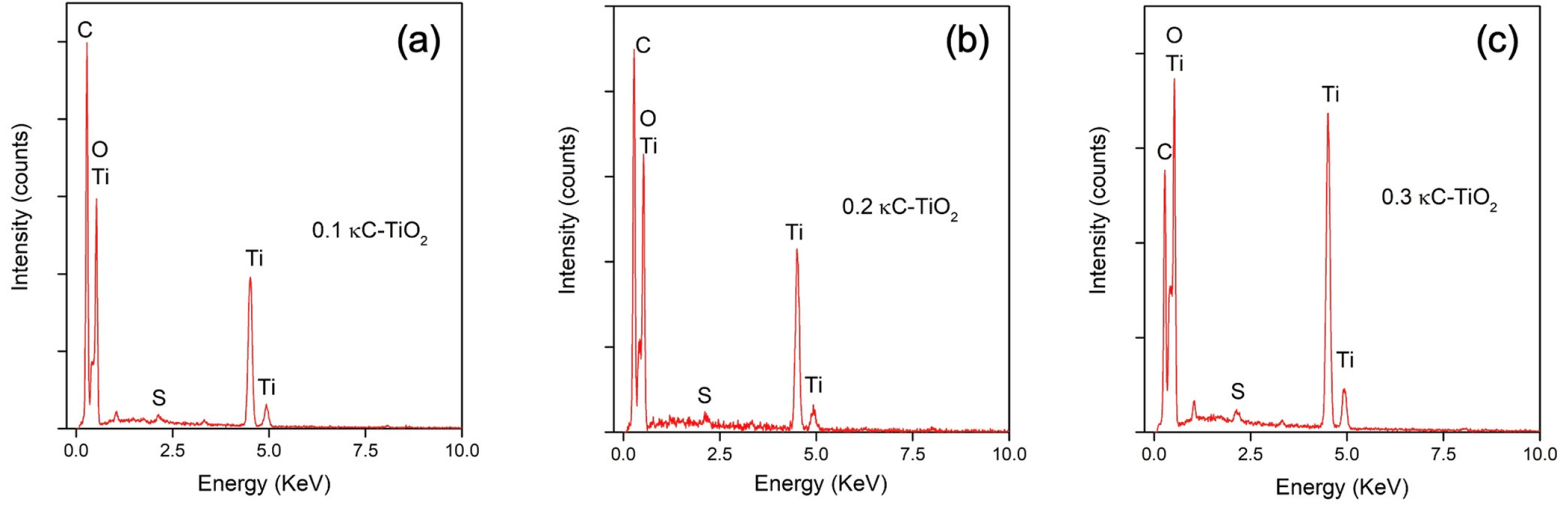
Energy-dispersive X-ray (EDX) spectrum of titanium dioxide nanoparticles (κC-TiO_2_ NPs) at different concentrations of kappa-carrageenan [0.1%, 0.2%, and 0.3%, respectively (a,b,c)].

To further understand the characteristics of the κC-TiO_2_ NPs, an EDX analysis was conducted. The EDX pattern of the codoped TiO_2_ shows the presence of the dopants C and S in the sample, as shown in Figure 8. The distinctive C–S codoped TiO_2_ spectra reveal the presence of Ti, O, C, and S elements. EDX analysis results indicated prominently intense peaks corresponding to Ti, alongside oxygen peaks, likely arising from dissociation of the precursor compound (titanium tetrabutoxide) or degraded κ-carrageenan employed in synthesis.

### Mechanism for κ-Carrageenan-Mediated Synthesis of TiO_2_ NPs

**Figure 9:**
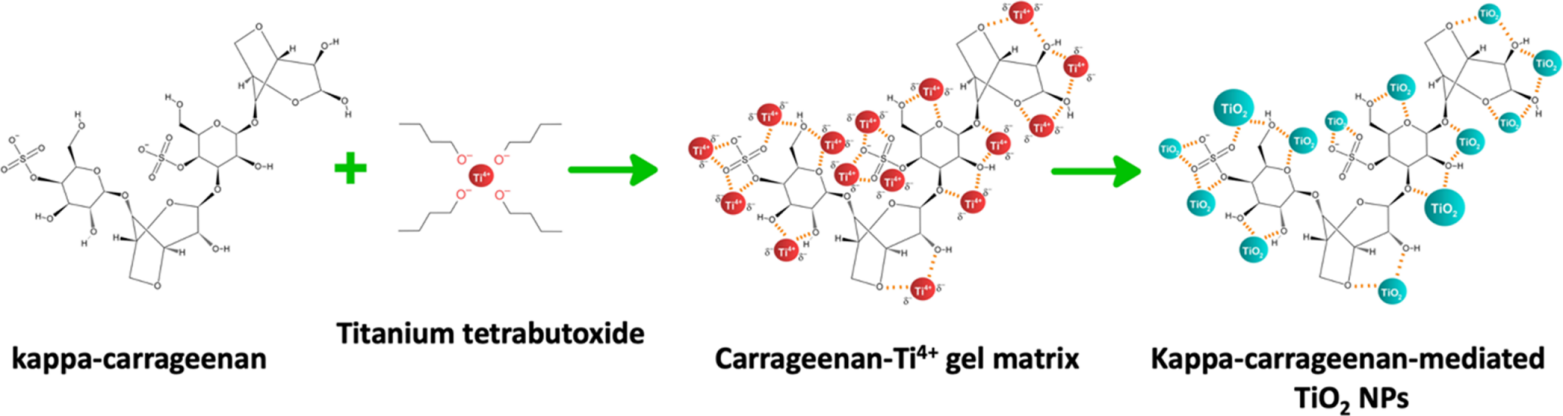
Proposed mechanism of the kappa-carrageenan-mediated synthesis of titanium dioxide nanoparticle (κC-TiO_2_ NPs) synthesis.

A sequential process can be delineated in synthesizing κ-carrageenan-mediated κC-TiO_2_ NPs, consisting of three fundamental phases: reduction, growth, and stabilization.^62^ The process of synthesizing TiO_2_ nanoparticles through the mediation of κ-carrageenan can be understood by examining the phases illustrated in Figure 9. First, Ti^4+^ (red circles) ions are reduced in the reduction phase, and the resultant Ti atoms initiate nucleation. In this crucial stage, the retrieval of Ti^4+^ ions occurs by interacting with κ-carrageenan from their salt precursor, titanium butoxide. The vinyl sulfonic acid groups on κ-carrageenan facilitate the absorption of Ti^4+^ ions from the solution, enabling the anchoring of Ti ions to the −SO_3_- functional groups within the hydrogel network. The hydroxyl groups (OH^–^) in the metabolites contribute to the reduction process by donating electrons, transforming Ti metal ions to a zerovalent state from a +4 oxidation state. Subsequently, these reduced titanium atoms undergo nucleation. Afterward, during the growth stage, small Ti nanoparticles naturally merge into larger ones through Ostwald ripening. This is facilitated by the greater binding energy among Ti metal atoms compared to atom-solvent interactions, thereby increasing the thermodynamic stability of Ti nanoparticles. The final phase, stabilization, occurs as the nanoparticles assume their most energetically favorable conformation, heavily influenced by the κ-carrageenan’s ability to stabilize the metal oxide nanoparticles. κ-carrageenan effectively caps the Ti nanoparticles, preventing further aggregation. After subsequent drying and calcination processes, the resulting κC-TiO_2_ NPs (green circles) are obtained.

### Photocatalytic Activity of the Synthesized TiO_2_ NPs

**Figure 10:**
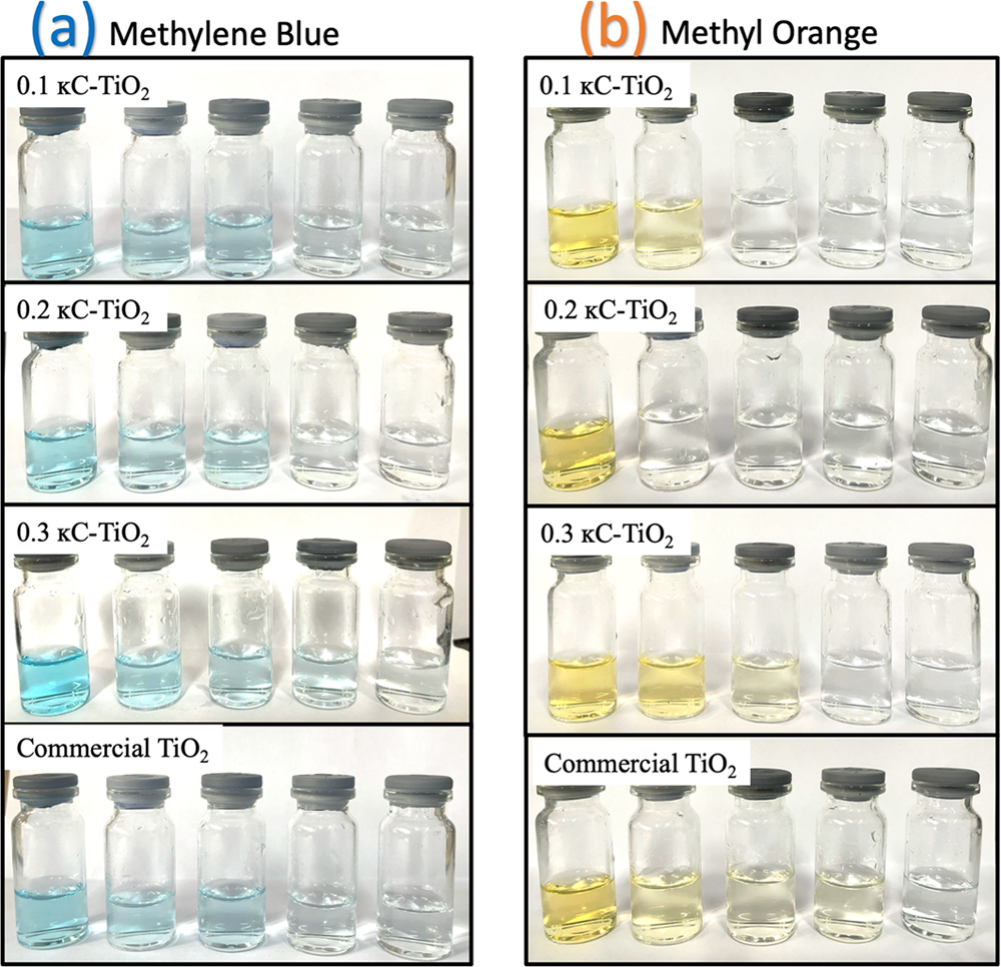
Color change illustration of (a) methylene blue and (b) methyl orange under different κC-TiO_2_ photocatalysts compared with pure commercial TiO_2_ NPs.

The study thoroughly investigates the photocatalytic activity of κC-TiO_2_ NPs synthesized using κ-carrageenan as a reducing and stabilizing agent. Specifically, this investigation concentrates on their effectiveness in degrading methylene blue (MB) and methyl orange (MO) when exposed to UV light irradiation. These two organic dyes were selected for their noticeable color changes (Figure 10) before and after degradation, aiding in the confirmation of the reactions.

**Figure 11:**
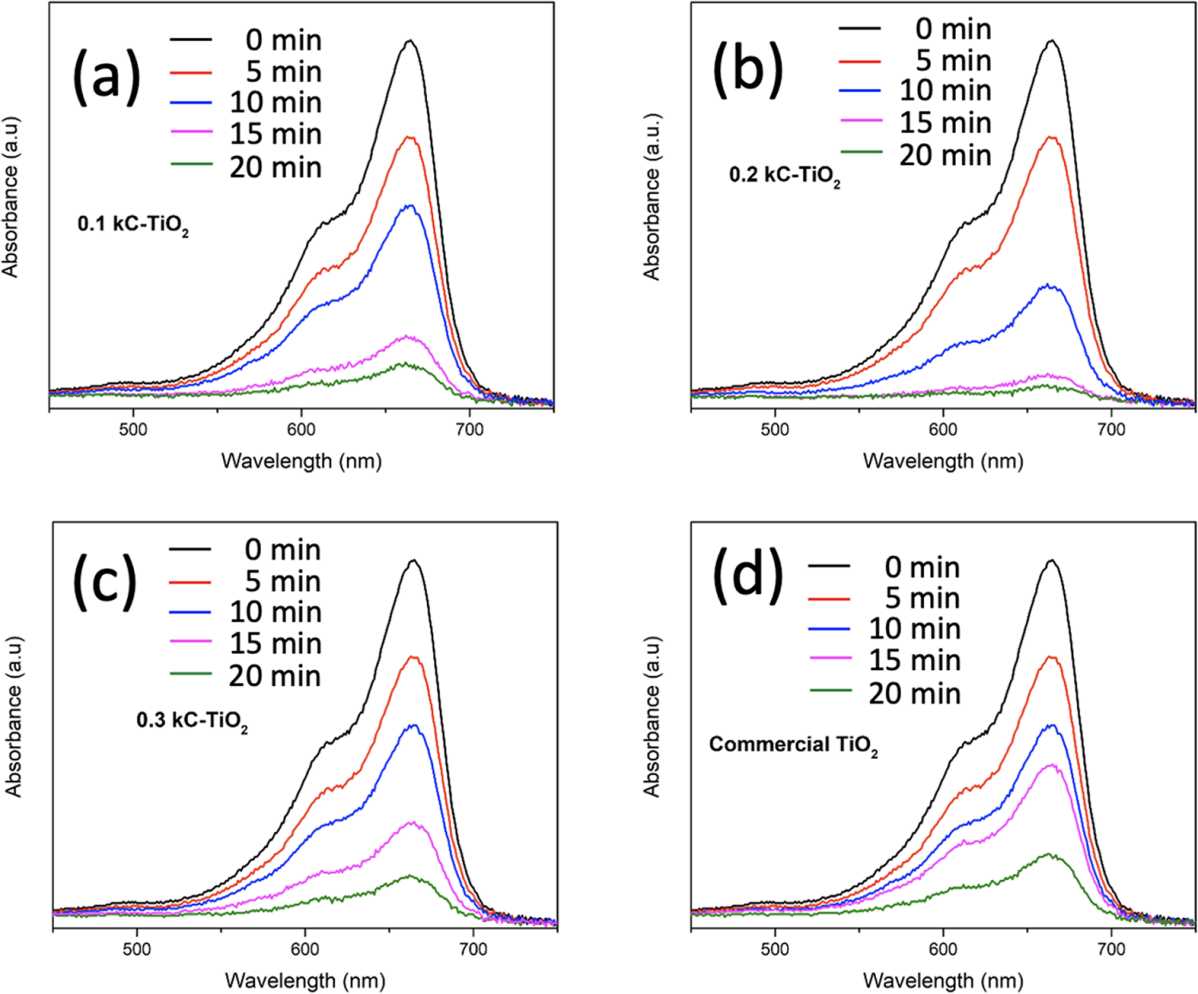
Photocatalytic activities of TiO_2_ nanoparticles synthesized using different concentrations of κ-carrageenan at (a) 0.1%, (b) 0.2%, and (c) 0.3% compared with (d) pure commercial TiO_2_ NPs for the degradation of MB dye.

The photocatalytic efficacies of the synthesized κC-TiO_2_ NPs were assessed for the degradation of MB dye when subjected to UV light irradiation (λ_max_ < 400 nm) in dark conditions. Samples were gathered at consistent time intervals and subsequently tested in UV–visible absorbance spectra spanning from 200 to 800 nm. The prominent absorption band at 664 nm corresponds to the maximum wavelength of MB dye, revealing a discernible decline in intensity over time (Figure 11) in the presence of κC-TiO_2_ NPs as the photocatalyst. The degradation kinetics were evaluated using the following equation (eq 3).
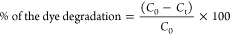
3where C_0_ denotes the initial concentration of the dye, and C_t_ represents the dye concentration at time “t” after UV irradiation. Figure 10a shows the decolorization observed for MB. It can be observed that 0.2 κC-TiO_2_ NP optically transformed the dye solution transparent at the fourth cycle compared with the other setups. This aligns with the specific degradation values found in Table 2. The experimental findings reveal a remarkable 99.47% degradation of MB dye under UV irradiation for 0.2 κC-TiO_2_ NPs photocatalyst. This is depicted in Figure 11b. Regarding 0.1 κC-TiO_2_ NPs, a substantial 93.36% degradation of MB was observed, depicted in Figure 11a. Conversely, the 0.3 κC-TiO_2_ NPs photocatalyst exhibited 90.77% MB dye degradation, as represented in Figure 11c. Commercial TiO_2_ displayed an 85.30% degradation rate, as evidenced in Figure 11d. Notably, 0.2 κC-TiO_2_ NPs demonstrated the most rapid color transformation.

**Table 2:** Percentage of Degradation for MB and MO at Different TiO_2_ NP Photocatalysts

	Degradation Efficiency (%)	
Sample	Methylene Blue (MB)	Methyl Orange (MO)
0.1 κC-TiO_2_	93.36	89.94
0.2 κC-TiO_2_	99.47	97.84
0.3 κC-TiO_2_	90.77	83.24
Commercial TiO_2_	85.30	74.56

**Figure 12:**
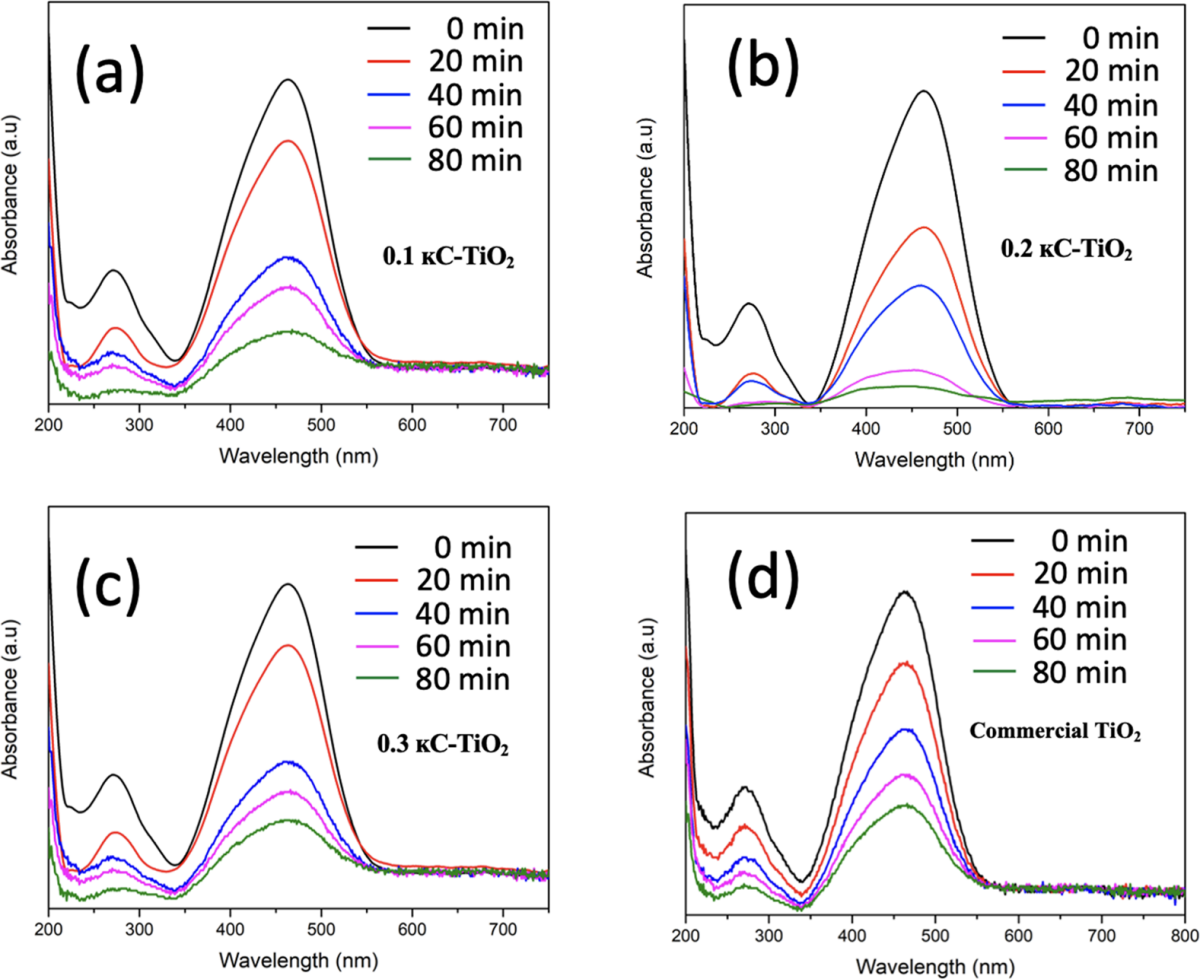
Photocatalytic activities of TiO_2_ nanoparticles synthesized using different concentrations of κ-carrageenan at (a) 0.1%, (b) 0.2%, and (c) 0.3%, compared with (d) pure commercial TiO_2_ NPs for the degradation of MO dye.

Comparable trends were noted in the photodegradation of MO. The photocatalyst 0.2 κC-TiO_2_ NPs demonstrated the swiftest degradation of MO at 97.84%, depicted in Figure 12b. This corresponds to the rapid disappearance of color at only the second cycle in Figure 10. Following closely, 0.1 κC-TiO_2_ NPs exhibited an 89.94% degradation of MO, as shown in Figure 12a. The 0.3 κC-TiO_2_ NPs sample displayed an 83.24% degradation of MO, illustrated in Figure 12c, while commercial TiO_2_ exhibited a 74.56% degradation, as demonstrated in Figure 12d. These results collectively underscore the efficiency of TiO_2_ photocatalysts, with 0.2 κC-TiO_2_ consistently outperforming other variants in both MB and MO degradation processes under UV irradiation conditions.

### Photodegradation of MB and MO Dye Using a 0.2 κC-TiO_2_ NP Photocatalyst

**Figure 13:**
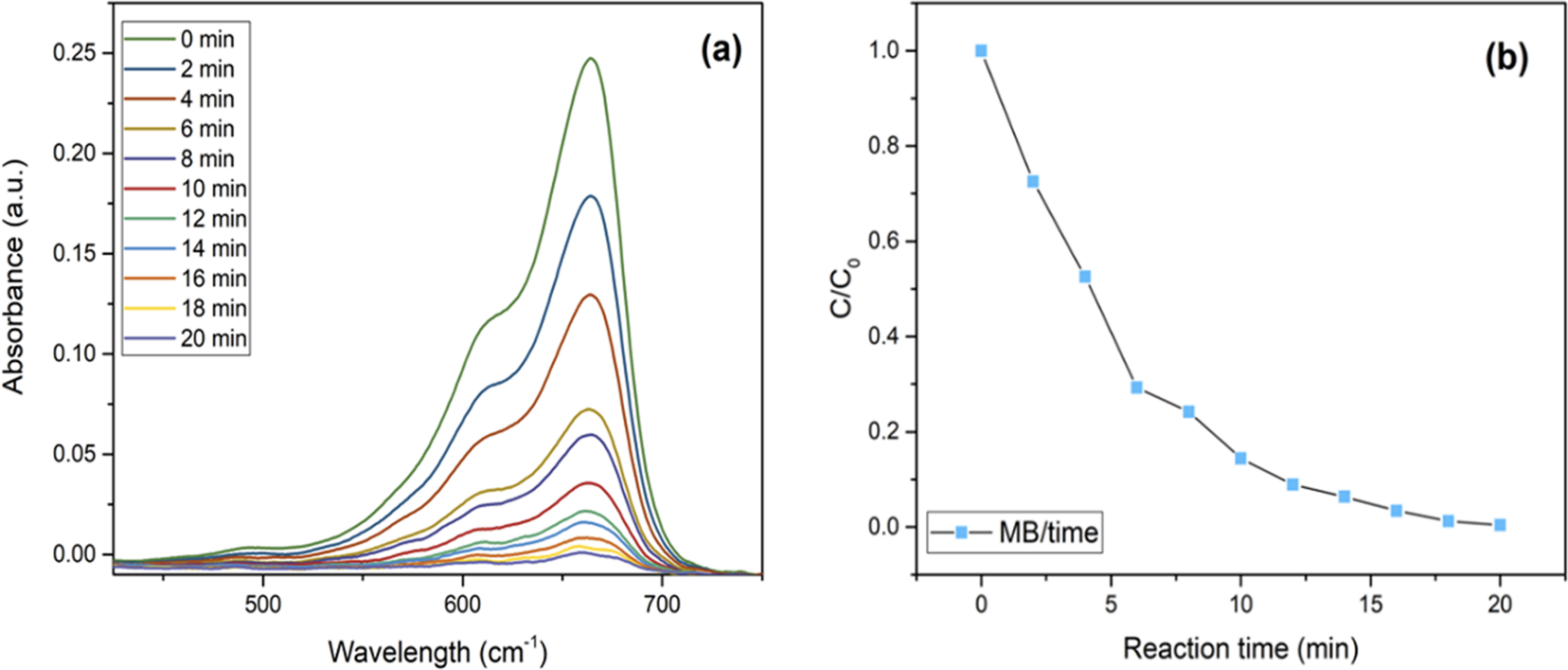
(a) Photocatalytic activity of the synthesized kappa-carrageenan-mediated titanium dioxide nanoparticles (κC-TiO_2_ NPs) was assessed for their photocatalytic activity in degrading methylene blue (MB) and (b) the kinetic behavior of MB photocatalytic degradation under UV lamp irradiation (λ *<* 400 nm) employing κC-TiO_2_ NPs.

The UV–vis spectrum analysis of MB provides valuable insights into the kinetics and mechanisms of its degradation. MB exhibits characteristic absorption bands for both oxidized and reduced states. The reduction of MB to leuco MB (LMB) can be quantified by tracking the reduction in absorption in the UV–visible spectrum at 664 nm.^63^ The time-dependent electronic absorption spectrum of MB under UV light exposure (Figures 13a and 13b) demonstrates the minimal reduction in the absence of the biosynthesized nanocatalyst (dye degradation 1.8%). However, in the presence of κC-TiO_2_, particularly those synthesized with 0.2% κ-carrageenan, a remarkable degradation of MB occurs, with over 99% achieved after just 20 min of UV light exposure. This noteworthy efficiency underscores the outstanding photocatalytic properties of κC-TiO_2_ NPs. Notably, no new absorption bands emerged during degradation, confirming the absence of stable reaction intermediates. This observation highlights that the degradation process proceeds directly from MB to its final degraded products, ensuring the eco-friendly removal of hazardous organic dyes, a crucial advancement in wastewater treatment and pollution control.

**Figure 14:**
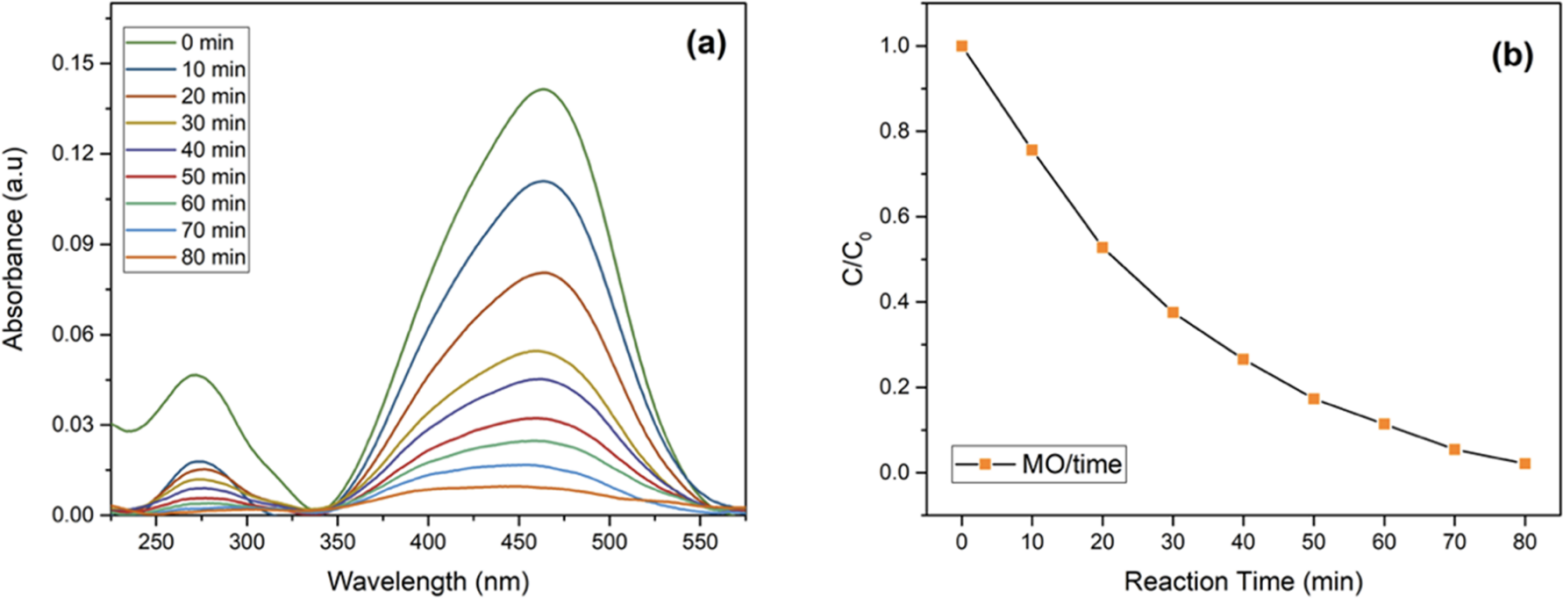
(a) Photocatalytic activity of the synthesized kappa-carrageenan-mediated titanium dioxide nanoparticles (κC-TiO_2_ NPs) assessed for their photocatalytic activity in degrading methyl orange (MO) and (b) the kinetic behavior of MO photocatalytic degradation under UV lamp irradiation (λ *<* 400 nm) employing κC-TiO_2_ NPs.

**Figure 15:**
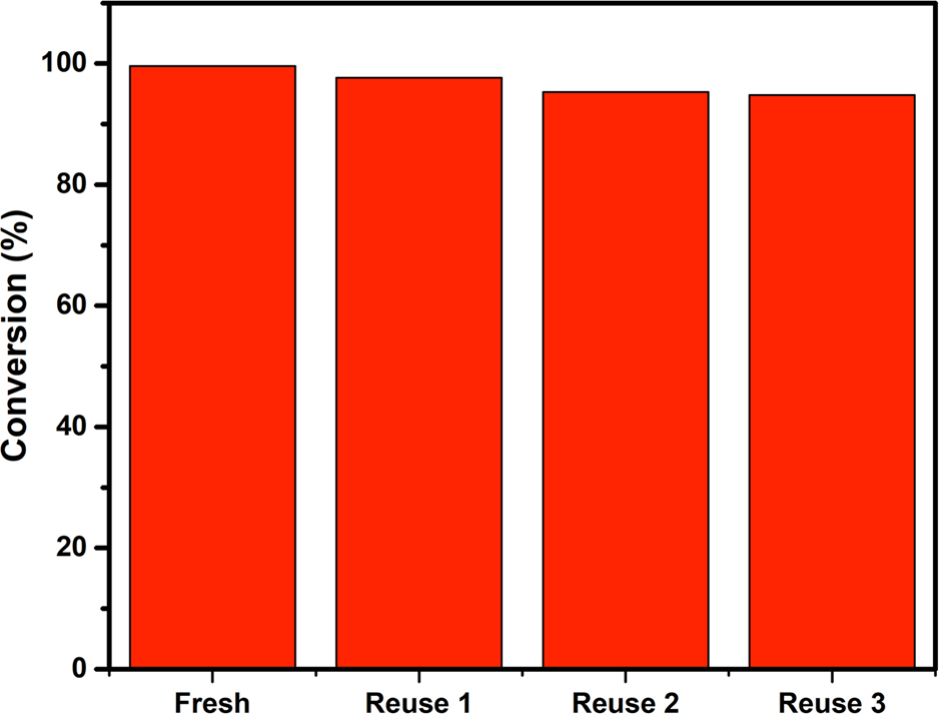
Reusability study of kappa-carrageenan (κ-carrageenan)-mediated titanium dioxide nanoparticles (κC-TiO_2_ NPs) for the degradation of methylene blue (MB) conducted under consistent reaction conditions (room temperature, 20 min reaction time).

The study also investigates the degradation of MO, known for its characteristic UV–visible absorption bands at 464 and 272 nm. These bands are linked to the azo bond (−N=N−) and the phenyl ring of MO, respectively.^64^ The reduction in these absorption bands during photocatalytic degradation reflects the cleavage of azo bonds and phenyl rings, leading to the mineralization and degradation of MO. Figures 14a and 14b depict the time-dependent changes in the absorption spectrum of MO during visible light exposure with the existence of κC-TiO_2_ NPs. Remarkably, within just 80 min of photoirradiation, κC-TiO_2_ NPs achieve a 97% degradation of MO. This underscores the potency of κC-TiO_2_ NPs in degrading complex organic dyes like MO, emphasizing their practical utility in environmental remediation. Indeed, the photocatalytic activity of κC-TiO_2_ is comparable to or slightly better than other TiO_2_-based photocatalysts reported in the literature (Table 3). After completing five cycles, it was observed that the percentage of MB dye degradation reached 94.8%, closely approaching the 99% achieved in the initial cycle. This finding validates the catalyst’s high photostability during repetitive reactions, as illustrated in the reusability study in Figure 15. The marginal reduction in degradation percentage could be ascribed to the catalyst loss during collection in each step.

**Table 3:** Performance of the Titanium Dioxide Nanoparticles Synthesized with Kappa-Carrageenan (κC-TiO_2_ NPs) as a Photocatalyst in Dye Degradation Compared to Other TiO_2_-Based Photocatalysts

s. no	photocatalyst type	preparation method	dye type	reaction time (min)	degradation	literature
1	TiO_2_	Biomediated	MB	120	92%	(50)
2	TiO_2_	Chemical	MB	120	60%	(51)
3	TiO_2_	Green synthesized	MB	120	96%	(52)
4	TiO_2_	Green synthesized	MB	60	95%	(53)
5	TiO_2_	Green synthesized	MB	20	99%	in this study
6	TiO_2_–Sn	Sol–gel method	MO	180	90%	(54)
7	TiO_2_	Green synthesized	MO	180	95%	(53)
8	TiO_2_/Ag	Hydrothermal	MO	120	65%	(55)
9	TiO_2_	Hydrothermal	MO	180	55%	(56)
10	TiO_2_	Green synthesized	MO	80	97%	In this study

### Mechanism of the Reduction of Band Gap

Nonmetal doping significantly contributes to boosting the photocatalytic efficiency of TiO_2_ by modifying its band structure. In the typical photocatalytic process of TiO_2_, illumination induces the excitation of valence electrons, leading to their migration from the valence band (VB) to the conduction band (CB) and the subsequent production of electron–hole pairs. During this process, a positive hole h^+^ is generated inside the valence band. The positive holes and liberated electrons undergo surface reactions on the photocatalyst in conjunction with water molecules that are adsorbed. Consequently, superoxide ions (•O_2_^–^) and •OH radicals are produced during the reaction, facilitating the degradation of MB and MO dyes.^65^

The overall reaction can be represented as follows:
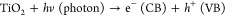
4
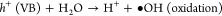
5
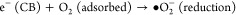
6
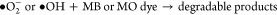
7

**Figure 16:**
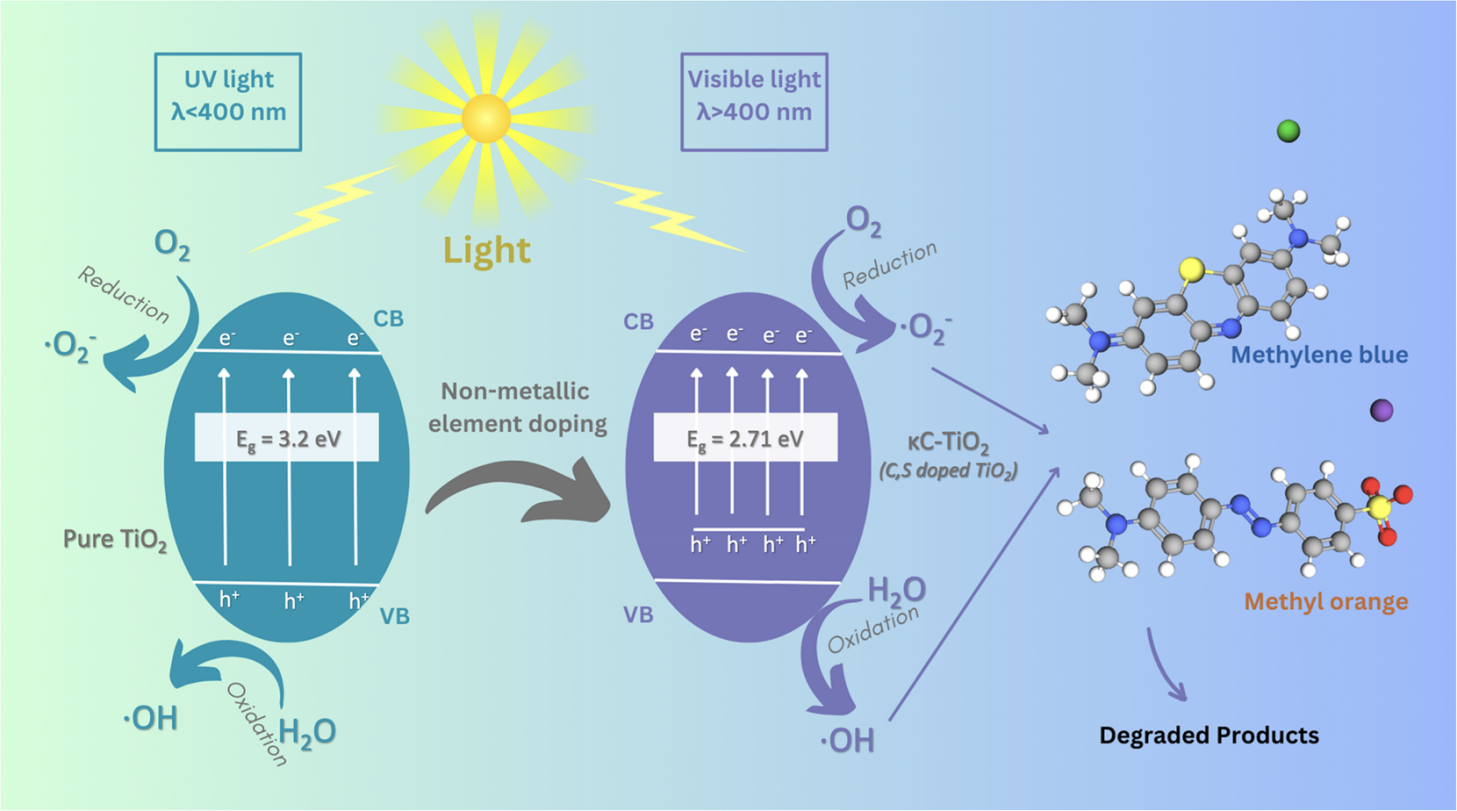
Mechanism of methylene blue and methyl orange degradation through photocatalysis employing a κC-TiO_2_ photocatalyst with a reduced band gap.

To increase the photoresponse spectrum of TiO_2_, nonmetal ion doping was employed, particularly carbon and sulfur sourced from κ-carrageenan, to replace oxygen or partial oxygen in TiO_2_ with nonmetals.^66^ This process reconstructs the VB of TiO_2_, shifting it upward and effectively reducing the band gap width (Figure 16). Consequently, the narrowed band gap allows TiO_2_ to absorb a broader spectrum of light, thereby increasing the utilization efficiency of sunlight. Prior research has also highlighted that the inclusion of graphitic carbon results in an expanded surface area, thereby amplifying the adsorption and light absorption capabilities of sulfur-doped TiO_2_ nanocatalysts.^39^ Furthermore, the modified band structure resulting from nonmetallic doping facilitates the effective disjunction of photoinduced electron–hole pairs. This separation is crucial for preventing recombination, as it impedes the loss of photoinduced charges before they can participate in the degradation reactions. The extended lifetime of these charge carriers enhances the availability of electrons and holes for redox reactions, such as the formation of •OH from water molecules.^67^ Notably, the synergistic effect of a reduced band gap, efficient charge separation, and increased light absorption, especially with dopants like sulfur and carbon, results in a substantial increase in the photocatalytic degradation rate of pollutants. The improved photodegradation of model contaminants like MO and MB exemplifies this enhanced performance.

## Conclusion

In this study, κ-carrageenan proved to be a versatile agent in synthesizing TiO_2_ NPs with enhanced photocatalytic activity. Varying concentrations of κ-carrageenan led to reduced band gaps and improved crystallinity in κC-TiO_2_ NPs, as confirmed by XRD analysis, showcasing highly crystalline anatase-phase particles without contaminants. UV–visible spectroscopic analysis indicated a lowered band gap energy in κC-TiO_2_ NPs in contrast to pure TiO_2_ NPs, attributed to the incorporation of residual carbon and sulfur from κ-carrageenan. SEM and TEM morphological analysis revealed that 0.2 κC-TiO_2_ NPs exhibited the most uniformly distributed particle size. These nanoparticles, particularly the 0.2 κC-TiO_2_ NPs, demonstrated superior photocatalytic efficiency in degrading MB and MO dyes under UV light, outperforming commercial TiO_2_ NPs. The study showcased the eco-friendly nature of κC-TiO_2_ NPs in repeated cycles of photocatalysis with high photostability, suggesting κ-carrageenan as a promising candidate for the sustainable synthesis of TiO_2_ NPs with enhanced photocatalytic properties, applicable in wastewater treatment and environmental remediation.

## Experimental Section

### Materials

Titanium(IV) butoxide [Ti(C_4_H_9_O)_4_, 97%], MB (C_16_H_18_C_l_N_3_S), MO (C_14_H_14_N_3_NaO_3_S), and various reagents were acquired from Sigma-Aldrich (USA) and utilized without undergoing additional purification. In every experiment, ultrapure water was utilized. κ-carrageenan was obtained from Niche Centers in the Region (NICER) R&D Seaweed Research and Development Center, Mindanao State University in Tawi–Tawi, Sanga–Sanga, Bongao, Tawi–Tawi, Philippines.

### Synthesis of TiO_2_ Using κ-Carrageenan

The solution of κ-carrageenan was created by dissolving 2.5 g of κ-carrageenan in 250 mL of ultrapure water, followed by thorough stirring for 30 min at 60 °C until complete dissolution. To examine the impact of κ-carrageenan concentration on the synthesis of TiO_2_ nanoparticles, three varying concentrations of κ-carrageenan (0.10%, 0.20%, and 0.30%) were chosen for investigation. Subsequently, a solution containing 0.4 M titanium butoxide was gradually introduced into the κ-carrageenan solution. The pH was adjusted to pH 9 through the incremental addition of 1 M NaOH. Vigorous stirring was maintained at 50 °C for 4.5 h. The resulting TiO_2_ NPs, produced with distinct κ-carrageenan concentrations, were left to age for 24 h at room temperature (25 °C). The final TiO_2_ NPs are produced through calcination at the temperature of 500 °C using a furnace for 2 h.

### Characterization of TiO_2_ NPs

XRD diffraction patterns of the powder were captured using a Cu Kα radiation source (40 kV and 30 mA) within a 3–60° 2θ range, employing 0.02° 2θ/0.60s on a Shimadzu XRD Maxima 7000 instrument from Japan. The phase of TiO_2_ was confirmed by comparing the prominent positions of reported peaks with those in the standard JCPDS database. UV spectroscopy was executed using a Genesys 10S UV–vis spectrophotometer from Thermo Fisher Scientific. Quartz cuvettes and ultrapure water as the reference solvent were employed for the analysis, and the absorption spectra were documented in the wavelength span of 200 to 800 nm. Fourier Transform Infrared (FTIR) spectroscopy, conducted using the Shimadzu FTIR-ATR IR Tracer-100 instrument from Tokyo, Japan, was utilized to analyze the samples and identify the specific functional groups within the spectral range of 4000–400 cm^–1^. The surface morphology of TiO_2_ NPs was scanned by scanning electron microscope (SEM) (JEOL, JSM IT200). TEM analysis was performed using a Jeol TEM system (JEM 2100 Plus LaB6 TEM with STEM). X-ray photoelectron spectroscopic (XPS) analysis was conducted using a JEOL JPS-9200 spectrometer (JEOL Ltd., Japan) equipped with a monochromatized Al Kα X-ray source operating at 100 W under ultrahigh vacuum (about 107 Pa). The narrow scan spectra of oxygen (O 1s), carbon (C 1s), titanium (Ti 2p), and sulfur (S 2p) were obtained and corrected using the binding energy of adventitious carbon (285.0 EV). All XPS spectra were deconvoluted with XPSPEAK version 4.1 using a true Shirley background and a 20–80% Lorentzian–Gaussian peak model.^68,69^ The specific surface area and pore size were analyzed using a BELSORP MAX X analysis instrument.

### Photocatalytic Degradation of Dyes

The degradation of methylene blue (MB) and methyl orange (MO) through photocatalysis involved the addition of 100 mg of TiO_2_ nanoparticles to 50 mL solutions containing 10^–3^ M concentrations of MB and MO, respectively, under stirring. After reaching adsorption–desorption equilibrium within 16 h, the mixture was exposed to irradiation from four (4) 10 W UV–B lamps (λ < 400 nm) positioned approximately 10 cm away. Continuous stirring persisted until the reaction was deemed complete, as evidenced by an alteration in the color of the solution. For the kinetic analysis of MB and MO degradation, samples were obtained at uniform intervals and examined via UV–vis spectroscopy. To assess the reusability of TiO_2_ NPs, photocatalytic degradation of MB was repeated three times under identical conditions. After each reaction, the catalyst was separated through centrifugation, washed with deionized water, and reused.

## References

[ref1] Vörösmarty C. J.; McIntyre P. B.; Gessner M. O.; Dudgeon D.; Prusevich A.; Green P.; Glidden S.; Bunn S. E.; Sullivan C. A.; Liermann C. R.; Davies P. M. Global Threats to Human Water Security and River Biodiversity. Nature 2010, 467 (7315), 555–561. 10.1038/nature09440. 20882010

[ref2] Halepoto H.; Gong T.; Memon H. Current Status and Research Trends of Textile Wastewater Treatments—A Bibliometric-Based Study. Front Environ. Sci. 2022, 10, 1–18. 10.3389/fenvs.2022.1042256.

[ref3] Mani S.; Chowdhary P.; Bharagava R. N. Textile Wastewater Dyes: Toxicity Profile and Treatment Approaches. In Emerging and Eco-Friendly Approaches for Waste Management; Springer: Singapore, 2018; pp 219–244. 10.1007/978-981-10-8669-4_11.

[ref4] Chowdhary P.; Bharagava R. N.; Mishra S.; Khan N. Role of Industries in Water Scarcity and Its Adverse Effects on Environment and Human Health. In Environmental Concerns and Sustainable Development; Springer: Singapore, 2020; pp 235–256. 10.1007/978-981-13-5889-0_12.

[ref5] Phugare S. S.; Kalyani D. C.; Surwase S. N.; Jadhav J. P. Ecofriendly Degradation, Decolorization and Detoxification of Textile Effluent by a Developed Bacterial Consortium. Ecotoxicol Environ. Saf 2011, 74 (5), 1288–1296. 10.1016/j.ecoenv.2011.03.003. 21524793

[ref6] Alsukaibi A. K. D. Various Approaches for the Detoxification of Toxic Dyes in Wastewater. Processes 2022, 10, 1968 10.3390/pr10101968.

[ref7] Rehman K.; Shahzad T.; Sahar A.; Hussain S.; Mahmood F.; Siddique M. H.; Siddique M. A.; Rashid M. I. Effect of Reactive Black 5 Azo Dye on Soil Processes Related to C and N Cycling. Peer J. 2018, 6 (5), e4802 10.7717/peerj.4802. 29844965 PMC5969049

[ref8] Al-Tohamy R.; Ali S. S.; Li F.; Okasha K. M.; Mahmoud Y. A.-G.; Elsamahy T.; Jiao H.; Fu Y.; Sun J. A Critical Review on the Treatment of Dye-Containing Wastewater: Ecotoxicological and Health Concerns of Textile Dyes and Possible Remediation Approaches for Environmental Safety. Ecotoxicology and Environmental Safety 2022, 231, 113160 10.1016/j.ecoenv.2021.113160. 35026583

[ref9] Bal G.; Thakur A. Distinct Approaches of Removal of Dyes from Wastewater: A Review Selection and Peer-Review under Responsibility of the Scientific Committee of the 2nd International Con-Ference on Functional Material, Manufacturing and Performances. Materials Today: Proceedings 2022, 50, 1575 10.1016/j.matpr.2021.09.119.

[ref10] Wong S.; Ghafar N. A.; Ngadi N.; Razmi F. A.; Inuwa I. M.; Mat R.; Amin N. A. S. Effective Removal of Anionic Textile Dyes Using Adsorbent Synthesized from Coffee Waste. Sci Rep 2020, 10.1038/s41598-020-60021-6. PMC7031400 32076087

[ref11] Golob V.; Vinder A.; Simonic M. Efficiency of the Coagulation/Flocculation Method for the Treatment of Dyebath Effluents. Dyes and Pigments 2005, 67, 93 10.1016/j.dyepig.2004.11.003.

[ref12] Raut P.; Pal D.; Singh V. K. Dye Removal Using Activated Sludge. Biological Approaches in Dye-Containing Wastewater 2022, 1–16. 10.1007/978-981-19-0526-1_1.

[ref13] Crini G.; Lichtfouse E. Advantages and Disadvantages of Techniques Used for Wastewater Treatment. Environmental Chemistry Letters; Springer Verlag, 2019; pp 145–155. 10.1007/s10311-018-0785-9.

[ref14] Leaper S.; Abdel-Karim A.; Gad-Allah T. A.; Gorgojo P. Air-Gap Membrane Distillation as a One-Step Process for Textile Wastewater Treatment. Chemical Engineering Journal 2019, 360, 1330–1340. 10.1016/j.cej.2018.10.209.

[ref15] Andreozzi R.; Caprio V.; Insola A.; Marotta R. Advanced Oxidation Processes (AOP) for Water Purification and Recovery. Catal. Today 1999, 53 (1), 51–59. 10.1016/S0920-5861(99)00102-9.

[ref16] Lee C.; Yoon J.; Von Gunten U. Oxidative Degradation of N-Nitrosodimethylamine by Conventional Ozonation and the Advanced Oxidation Process Ozone/Hydrogen Peroxide. Water Res. 2007, 41 (3), 581–590. 10.1016/j.watres.2006.10.033. 17184813

[ref17] Gaya U. I.; Abdullah A. H. Heterogeneous Photocatalytic Degradation of Organic Contaminants over Titanium Dioxide: A Review of Fundamentals, Progress and Problems. Journal of Photochemistry and Photobiology C: Photochemistry Reviews 2008, 9 (1), 1–12. 10.1016/j.jphotochemrev.2007.12.003.

[ref18] Al-Nuaim M. A.; Alwasiti A. A.; Shnain Z. Y. The Photocatalytic Process in the Treatment of Polluted Water. Chemical Papers 2022 77:2 2023, 77 (2), 677–701. 10.1007/s11696-022-02468-7. PMC9527146 36213320

[ref19] Tanwar N.; Dhiman V.; Kumar S.; Kondal N. Plant Extract Mediated ZnO-NPs as Photocatalyst for Dye Degradation: An Overview. Mater. Today Proc. 2022, 48, 1401–1406. 10.1016/j.matpr.2021.09.186.

[ref20] Aroob S.; Carabineiro S. A. C.; Taj M. B.; Bibi I.; Raheel A.; Javed T.; Yahya R.; Alelwani W.; Verpoort F.; Kamwilaisak K.; Al-Farraj S.; Sillanpää M. Green Synthesis and Photocatalytic Dye Degradation Activity of CuO Nanoparticles. Catalysts 2023, 13, 502 10.3390/catal13030502.

[ref21] Rathi V. H.; Jeice A. R. Green Fabrication of Titanium Dioxide Nanoparticles and Their Applications in Photocatalytic Dye Degradation and Microbial Activities. Chemical Physics Impact 2023, 6, 100197 10.1016/j.chphi.2023.100197.

[ref22] Far H.; Hamici M.; Brihi N.; Haddadi K.; Boudissa M.; Chihi T.; Fatmi M. High-Performance Photocatalytic Degradation of NiO Nanoparticles Embedded on α-Fe_2_O_3_ Nanoporous Layers under Visible Light Irradiation. Journal of Materials Research and Technology 2022, 19, 1944–1960. 10.1016/j.jmrt.2022.05.159.

[ref23] Kolokolov D. S.; Podurets A. A.; Nikonova V. D.; Vorontsov-Velyaminov P. N.; Bobrysheva N. P.; Osmolowsky M. G.; Osmolovskaya O. M.; Voznesenskiy M. A. SnO_2_ Nanoparticles with Different Aspect Ratio and Structural Parameters: Fabrication, Photocatalytic Efficiency Dependences and Fast Organic Dyes Degradation. Appl. Surf. Sci. 2022, 599, 153943 10.1016/j.apsusc.2022.153943.

[ref24] Fujishima A.; Honda K. Electrochemical Photolysis of Water at a Semiconductor Electrode. Nature 1972, 238, 37 10.1038/238037a0. 12635268

[ref25] Lee S. Y.; Park S. J. TiO_2_ Photocatalyst for Water Treatment Applications. Journal of Industrial and Engineering Chemistry 2013, 19, 1761–1769. 10.1016/j.jiec.2013.07.012.

[ref26] Wei Y.; Meng H.; Wu Q.; Bai X.; Zhang Y. TiO_2_-Based Photocatalytic Building Material for Air Purification in Sustainable and Low-Carbon Cities: A Review. Catalysts 2023, 13, 1466 10.3390/catal13121466.

[ref27] Yang H.; Yang B.; Chen W.; Yang J. Preparation and Photocatalytic Activities of TiO_2_-Based Composite Catalysts. Catalysts 2022, 12, 1263 10.3390/catal12101263.

[ref28] Hoffmann M. R.; Martin S. T.; Choi W.; Bahnemann D. W.; Keck W. M. Environmental Applications of Semiconductor Photocatalysis. Chem. Rev. 1995, 95, 69 10.1021/cr00033a004.

[ref29] Gomes J.; Lincho J.; Domingues E.; Quinta-Ferreira R. M.; Martins R. C. N-TiO_2_ Photocatalysts: A Review of Their Characteristics and Capacity for Emerging Contaminants Removal. Water (Switzerland) 2019, 11, 373 10.3390/w11020373.

[ref30] Alharshan G. A.; Aboraia A. M.; Uosif M. A. M.; Sharaf I. M.; Shaaban E. R.; Saad M.; ALMohiy H.; Elsenety M. M. Optical Band Gap Tuning, DFT Understandings, and Photocatalysis Performance of ZnO Nanoparticle-Doped Fe Compounds. Materials 2023, 16 (7), 2676 10.3390/ma16072676. 37048969 PMC10096406

[ref31] Sen P.; Bhattacharya P.; Mukherjee G.; Ganguly J.; Marik B.; Thapliyal D.; Verma S.; Verros G. D.; Chauhan M. S.; Arya R. K. Advancements in Doping Strategies for Enhanced Photocatalysts and Adsorbents in Environmental Remediation. Technologies; Multidisciplinary Digital Publishing Institute (MDPI); October 1, 2023. 10.3390/technologies11050144.

[ref32] Khedr T. M.; El-Sheikh S. M.; Hakki A.; Ismail A. A.; Badawy W. A.; Bahnemann D. W. Highly Active Non-Metals Doped Mixed-Phase TiO_2_ for Photocatalytic Oxidation of Ibuprofen under Visible Light. J. Photochem. Photobiol. A Chem. 2017, 346, 530–540. 10.1016/j.jphotochem.2017.07.004.

[ref33] Jalalah M.; Faisal M.; Bouzid H.; Ismail A. A.; Al-Sayari S. A. Dielectric and Photocatalytic Properties of Sulfur Doped TiO2 Nanoparticles Prepared by Ball Milling. Mater. Res. Bull. 2013, 48 (9), 3351–3356. 10.1016/j.materresbull.2013.05.023.

[ref34] Negi C.; Kandwal P.; Rawat J.; Sharma M.; Sharma H.; Dalapati G.; Dwivedi C. Carbon-Doped Titanium Dioxide Nanoparticles for Visible Light Driven Photocatalytic Activity. Appl. Surf. Sci. 2021, 554, 149553 10.1016/j.apsusc.2021.149553.

[ref35] Rupert R.; Rodrigues K. F.; Thien V. Y.; Yong W. T. L. Carrageenan From Kappaphycus Alvarezii (Rhodophyta, Solieriaceae): Metabolism, Structure, Production, and Application. Frontiers in Plant Science; Frontiers Media S.A., May 10, 2022. 10.3389/fpls.2022.859635. PMC9127731 35620679

[ref36] Rochas C.; Rinaudo M. Mechanism of Gel Formation in K-Carrageenan. Biopolymers 1984, 23, 735 10.1002/bip.360230412.

[ref37] Wan H.; Li C.; Mahmud S.; Liu H. Kappa Carrageenan Reduced-Stabilized Colloidal Silver Nanoparticles for the Degradation of Toxic Azo Compounds. Colloids Surf. A Physicochem Eng. Asp 2021, 616, 126325 10.1016/j.colsurfa.2021.126325.

[ref38] Michel G.; Chantalat L.; Fanchon E.; Henrissat B.; Kloareg B.; Dideberg O. The ι-Carrageenase of Alteromonas Fortis: A β-Helix Fold-Containing Enzyme for the Degradation of a Highly Polyanionic Polysaccharide. J. Biol. Chem. 2001, 276 (43), 40202–40209. 10.1074/jbc.M100670200. 11493601

[ref39] Chaudhary J. P.; Mahto A.; Vadodariya N.; Kholiya F.; Maiti S.; Nataraj S. K.; Meena R. Fabrication of Carbon and Sulphur-Doped Nanocomposites with Seaweed Polymer Carrageenan as an Efficient Catalyst for Rapid Degradation of Dye Pollutants Using a Solar Concentrator. RSC Adv. 2016, 6 (66), 61716–61724. 10.1039/C6RA10317K.

[ref40] Palajonnala Narasaiah B.; Banoth P.; mante Dominguez A. G.; Mandal B. K.; Kumar C. K.; Barnes C. H. W.; De Los Santos Valladares L.; Kollu P. Biogenic Photo-Catalyst TiO_2_ Nanoparticles for Remediation of Environment Pollutants. ACS Omega 2022, 7, 26174 10.1021/acsomega.2c01763. 35936468 PMC9352162

[ref41] Cravanzola S.; Cesano F.; Gaziano F.; Scarano D. Sulfur-Doped TiO_2_: Structure and Surface Properties. Catalysts 2017, 7 (7), 214 10.3390/catal7070214. PMC5701632 29209606

[ref42] Jalalah M.; Faisal M.; Bouzid H.; Ismail A. A.; Al-Sayari S. A. Dielectric and Photocatalytic Properties of Sulfur Doped TiO_2_ Nanoparticles Prepared by Ball Milling. Mater. Res. Bull. 2013, 48 (9), 3351–3356. 10.1016/j.materresbull.2013.05.023.

[ref43] Joo J. B.; Zhang Q.; Dahl M.; Lee I.; Goebl J.; Zaera F.; Yin Y. Control of the Nanoscale Crystallinity in Mesoporous TiO2 Shells for Enhanced Photocatalytic Activity. Energy Environ. Sci. 2012, 5 (4), 6321–6327. 10.1039/C1EE02533C.

[ref44] Devikala S.; Abisharani J. M. Green Synthesis of TiO_2_ Nanoparticles Using Averrhoa Bilimbi Fruits Extract and DPT-PEG Polymer Electrolyte for Enhance Dye-Sensitized Solar Cell Application. Dyes and Pigments - Insights and Applications 2023, 10.5772/intechopen.106944.

[ref45] Saranya S. K. S. K. S.; Padil V. V. T.; Senan C.; Pilankatta R.; Saranya S. K. K.; George B.; Wacławek S.; Cerník M. Green Synthesis of High Temperature Stable Anatase Titanium Dioxide Nanoparticles Using Gum Kondagogu: Characterization and Solar Driven Photocatalytic Degradation of Organic Dye. Nanomaterials 2018, 8 (12), 1002 10.3390/nano8121002. 30518035 PMC6316888

[ref46] Piatkowska A.; Janus M.; Szymanski K.; Mozia S. C-,N-and S-Doped TiO_2_ Photocatalysts: A Review. Catalysts 2021, 11, 144 10.3390/catal11010144.

[ref47] Alarif W. M.; Shaban Y. A.; Orif M. I.; Ghandourah M. A.; Turki A. J.; Alorfi H. S.; Tadros H. R. Z. Green Synthesis of TiO_2_ Nanoparticles Using Natural Marine Extracts for Antifouling Activity. Mar Drugs 2023, 21 (2), 62 10.3390/md21020062. 36827102 PMC9962051

[ref48] Yu J. C.; Ho W.; Lin J.; Yip H.; Wong P. K. Photocatalytic Activity, Antibacterial Effect, and Photoinduced Hydrophilicity of TiO_2_ Films Coated on a Stainless Steel Substrate. Environ. Sci. Technol. 2003, 37 (10), 2296–2301. 10.1021/es0259483. 12785540

[ref49] Hussain S. T.; Khan K.; Hussain R. Size Control Synthesis of Sulfur Doped Titanium Dioxide (Anatase) Nanoparticles, Its Optical Property and Its Photo Catalytic Reactivity for CO_2_ + H_2_O Conversion and Phenol Degradation. Journal of Natural Gas Chemistry 2009, 18 (4), 383–391. 10.1016/S1003-9953(08)60133-4.

[ref50] Elsupikhe R. F.; Shameli K.; Ahmad B.; Ibrahim A.; Zainudin N. Green Sonochemical Synthesis of Silver Nanoparticles at Varying Concentrations of κ-Carrageenan. Nanoscale Res Lett 2015, 10.1186/s11671-015-0916-1. PMC4523502 26220106

[ref51] Hug S. J. In Situ Fourier Transform Infrared Measurements of Sulfate Adsorption on Hematite in Aqueous Solutions.Jounal of Colloid and Interface Science 1997; Vol. 188.

[ref52] Wang Y.; Wang Y.; Meng Y.; Ding H.; Shan Y.; Zhao X.; Tang X. A Highly Efficient Visible-Light-Activated Photocatalyst Based on Bismuth- And Sulfur-Codoped TiO_2_. J. Phys. Chem. C 2008, 112 (17), 6620–6626. 10.1021/jp7110007.

[ref53] Jung S. M.; Grange P. TiO_2_-SiO_2_ Mixed Oxide Modified with H_2_SO_4_ II. Acid Properties and Their SCR Reactivity. Applied Catalysis A: General, 2002; Vol. 228.

[ref54] Zhu M.; Zhai C.; Qiu L.; Lu C.; Paton A. S.; Du Y.; Goh M. C. New Method to Synthesize S-Doped TiO_2_ with Stable and Highly Efficient Photocatalytic Performance under Indoor Sunlight Irradiation. ACS Sustain Chem. Eng. 2015, 3 (12), 3123–3129. 10.1021/acssuschemeng.5b01137.

[ref55] Zhang Y.; Huang Y.; Srot V.; van Aken P. A.; Maier J.; Yu Y. Enhanced Pseudo-Capacitive Contributions to High-Performance Sodium Storage in TiO_2_/C Nanofibers via Double Effects of Sulfur Modification. Nanomicro Lett. 2020, 10.1007/s40820-020-00506-1. PMC7770798 34138160

[ref56] Chen C.; Yang Y.; Ding S.; Wei Z.; Tang X.; Li P.; Wang T.; Cao G.; Zhang M. S-Doped Carbon@TiO_2_ to Store Li^+^/Na^+^ with High Capacity and Long Life-Time. Energy Storage Materials; 2018.

[ref57] Liu B.; Nakata K.; Sakai M.; Saito H.; Ochiai T.; Murakami T.; Takagi K.; Fujishima A. Hierarchical TiO_2_ Spherical Nanostructures with Tunable Pore Size, Pore Volume, and Specific Surface Area: Facile Preparation and High-Photocatalytic Performance. Catal. Sci. Technol. 2012, 2 (9), 1933–1939. 10.1039/c2cy00509c.

[ref58] Armaković S. J.; Savanović M. M.; Armaković S. Titanium Dioxide as the Most Used Photocatalyst for Water Purification: An Overview. Catalysts 2023, 13, 26 10.3390/catal13010026.

[ref59] Li D.; Song H.; Meng X.; Shen T.; Sun J.; Han W.; Wang X. Effects of Particle Size on the Structure and Photocatalytic Performance by Alkali-Treated TiO2. Nanomaterials 2020, 10 (3), 546 10.3390/nano10030546. 32197421 PMC7153365

[ref60] Vione D.; Minero C.; Maurino V.; Carlotti M. E.; Picatonotto T.; Pelizzetti E. Degradation of Phenol and Benzoic Acid in the Presence of a TiO_2_-Based Heterogeneous Photocatalyst. Appl. Catal., B 2005, 58 (1–2), 79–88. 10.1016/j.apcatb.2004.11.018.

[ref61] Varshney G.; Kanel S. R.; Kempisty D. M.; Varshney V.; Agrawal A.; Sahle-Demessie E.; Varma R. S.; Nadagouda M. N. Nanoscale TiO_2_ Films and Their Application in Remediation of Organic Pollutants. Coord. Chem. Rev. 2016, 306, 43–64. 10.1016/j.ccr.2015.06.011.

[ref62] Malik P.; Shankar R.; Malik V.; Sharma N.; Mukherjee T. K. Green Chemistry Based Benign Routes for Nanoparticle Synthesis. Journal of Nanoparticles 2014, 2014, 1–14. 10.1155/2014/302429.

[ref63] Kassale A.; Barouni K.; Bazzaoui M.; Albourine A. Kinetics and Modeling of the Adsorption of Methylene Blue by the Grafted Cotton; 2015; Vol. 5. www.jcbsc.org.

[ref64] Joseph S.; Mathew B. Microwave-Assisted Green Synthesis of Silver Nanoparticles and the Study on Catalytic Activity in the Degradation of Dyes. J. Mol. Liq. 2015, 204, 184–191. 10.1016/j.molliq.2015.01.027.

[ref65] Su Y.; Yang Y.; Zhang H.; Xie Y.; Wu Z.; Jiang Y.; Fukata N.; Bando Y.; Wang Z. L. Enhanced Photodegradation of Methyl Orange with TiO_2_ Nanoparticles Using a Triboelectric Nanogenerator. Nanotechnology 2013, 24 (29), 295401 10.1088/0957-4484/24/29/295401. 23807032

[ref66] Fang W.; Xing M.; Zhang J. Modifications on Reduced Titanium Dioxide Photocatalysts: A Review. Journal of Photochemistry and Photobiology C: Photochemistry Reviews 2017, 32, 21 10.1016/j.jphotochemrev.2017.05.003.

[ref67] Li R.; Li T.; Zhou Q. Impact of Titanium Dioxide (TiO_2_) Modification on Its Application to Pollution Treatment—a Review. Catalysts 2020, 10, 804 10.3390/catal10070804.

[ref68] Zoleta J.; Itao G.; Resabal V. J.; Lubguban A.; Corpuz R.; Tabelin C.; Ito M.; Hiroyoshi N. CeO_2_ -Dolomite as Fire Retardant Additives on the Conventional Intumescent Coating in Steel Substrate for Improved Performance. MATEC Web of Conferences 2019, 268, 04009 10.1051/matecconf/201926804009.

[ref69] Zoleta J. B.; Itao G. B.; Resabal V. J. T.; Lubguban A. A.; Corpuz R. D.; Ito M.; Hiroyoshi N.; Tabelin C. B. Improved Pyrolysis Behavior of Ammonium Polyphosphate-Melamine-Expandable (APP-MEL-EG) Intumescent Fire Retardant Coating System Using Ceria and Dolomite as Additives for I-Beam Steel Application. Heliyon 2020, 6 (1), e03119 10.1016/j.heliyon.2019.e03119. 31909279 PMC6940668

